# Classifying human promoters by occupancy patterns identifies recurring sequence elements, combinatorial binding, and spatial interactions

**DOI:** 10.1186/s12915-018-0585-5

**Published:** 2018-11-15

**Authors:** Xinyi Yang, Martin Vingron

**Affiliations:** Max Planck Institute for Molecular Genetics, 14195 Berlin, Germany, Ihnestraße 63-73, Berlin, 14195 Germany

**Keywords:** Promoters, Biclustering, Transcription factor combinatorics, Promoter-enhancer interaction

## Abstract

**Background:**

Characterizing recurring sequence patterns in human promoters has been a challenging undertaking even nowadays where a near-complete overview of promoters exists. However, with the more recent availability of genomic location (ChIP-seq) data, one can approach that question through the identification of characteristic patterns of transcription factor occupancy and histone modifications.

**Results:**

Based on the ENCODE annotation and integration of sequence motifs as well as three-dimensional chromatin data, we have undertaken a re-analysis of occupancy and sequence patterns in human promoters. We identify clear groups of CAAT-box and E-box sequence motif containing promoters, as well as a group of promoters whose interaction with an enhancer appears to be mediated by CCCTC-binding factor (CTCF) binding on the promoter. We also extend our analysis to inactive promoters, showing that only a surprisingly small number of inactive promoters is repressed by the polycomb complex. We also identify combinatorial patterns of transcription factor interactions indicated by the ChIP-seq signals.

**Conclusion:**

Our analysis defines subgroups of promoters characterized by stereotypic patterns of transcription factor occupancy, and combinations of specific sequence patterns which are required for their binding. This grouping provides new hypotheses concerning the assembly and dynamics of transcription factor complexes at their respective promoter groups, as well as questions on the evolutionary origin of these groups.

**Electronic supplementary material:**

The online version of this article (10.1186/s12915-018-0585-5) contains supplementary material, which is available to authorized users.

## Background

The sequence patterns which characterize mammalian promoters have been the object of intense study for many years (see, e.g., [1] for review). A number of sequence patterns, most prominently the TATA-Box, have been associated to promoters. At the same time, the wish to recognize promoters from sequence alone has prompted the search for further characteristic patterns. In the fruit fly, such patterns could indeed be delineated (see [2] and [3]). For mammalian promoters, a number of sequence patterns were identified as enriched based on careful sequence analysis (see, e.g., Bucher [4] and FitzGerald et al. [5, 6]). From amongst those patterns, the TATA-box used to be considered a hallmark of mammalian transcription. However, the more promoters became known the fewer of them actually contained a TATA-box. At the same time, CpG islands have been identified as an important feature of a human promoter (see [7] for review), even dividing the human promoters into the ones that possess a CpG island vs. the ones that lack it ([8, 9]).

The ENCODE project [10] has produced ample data on human promoter occupancy using the chromatin immunoprecipitation with massively parallel DNA sequencing (ChIP-seq) technology. It became clear that active promoters show particular histone modification patterns like an increased level of Histone 3 Lysine 4 trimethylation (H3K4me3) and of H3K27ac. Several papers exploited the data-set to improve on the definition of transcription factor binding sequences [11–13]. ENCODE data also comprises an extensive set of genomic location data for transcription factors and other chromatin-associated proteins, which do not bind in a sequence-specific manner. The group of Zhiping Weng and their collaborators already provided an in-depth analysis of the binding sites and their co-occurences in [14] and the associated factorbook-website [15]. However, their focus was on the binding sites in general and not on the promoters and their activity status. With a focus on the NF-Y transcription factor, Dolfini et al. studied the associated regulatory module [16]. Giannopoulou et al. [17] and Gerstein et al. [18] compiled a regulatory network and chromatin-bound protein complexes. Dan et al. studied TF colocalization. The question remains what can be learned from an integrated analysis of histone modifications and transcription factor occupancy patterns with respect to subgroups of promoters and their characteristic sequence patterns.

In this paper, we focus on the human promoter regions as defined in the RefSeq [19] database and integrate transcription factor binding information, histone modifications, and chromatin-associated proteins for the purpose of delineating subgroups of promoters. We collect promoter activity information in the form of CAGE tags [20] as reported by the FANTOM consortium. It is the RefSeq annotation that provides the static information that a chromosomal position can in principle act as a transcription start site, and the CAGE information that provides the dynamic information on promoter activity. As a control, we repeat the analysis with promoters identified from CAGE peaks only and independent of RefSeq annotation. From the ENCODE data, we process the ChIP-seq experiments for the histone marks (HMs) and transcription factors (TFs) for the two cell-lines with the largest available number of experiments (GM12878, K562). We will also verify our generated hypotheses in HeLa-cell data. For each cell-line, our input data constitutes a large matrix where a promoter defines a column, and each row corresponds to one ENCODE ChIP-seq experiment. For each column, the number of CAGE tags indicates transcriptional activity from this TSS and allows us to distinguish active and inactive promoters. Using the computational method of biclustering [21], we determine groups of promoters and associated groups of ChIP-seq experiments, where the promoters are occupied by just these TFs or HMs and, vice versa, the TFs/HMs are characteristic of these promoters. This information is visualized in a heatmap depiction of the matrix with columns and rows arranged in such a way that the groups become visible as blocks in the matrix. Note that biclustering a matrix differs from combining two hierarchical clusterings on either dimension in that biclustering selects combinations of groups of rows and groups of columns, together forming a homogeneous bicluster [22].

Analysis of the heatmap that results from biclustering uncovers several classes of promoters that are distinguished by the combinations of bound TFs. We will back up this information by motif analysis and, where meaningful, with chromatin 3D information. We analyze both active promoters and inactive promoters. Clustering information will be further supported by statistical tests for enrichment of occupancy and of motif occurrence. Taken together, our results demonstrate distinct subgroups of promoters defined by TF occupancy with the existence of the sequence patterns a prerequisite for the binding of the respective proteins.

## Results

### Sorting heatmaps for active and inactive promoters using biclustering

For each of the two cell-lines, GM12878 and K562, we study active and inactive promoters separately, dividing them based on the number of CAGE tags mapping to the promoters as proposed by FANTOM ([20], for details on this and the following steps see the “Methods” section). GM12878 yielded 6030 active genes and 6854 inactive genes, while K562 has 4172 active genes and 9588 inactive genes. For each promoter, we collected from the ENCODE data [10] the ChIP-seq read-counts for these promoter regions in the respective cell-lines, see the “Methods” section for processing of read-counts.

**Fig. 1.**
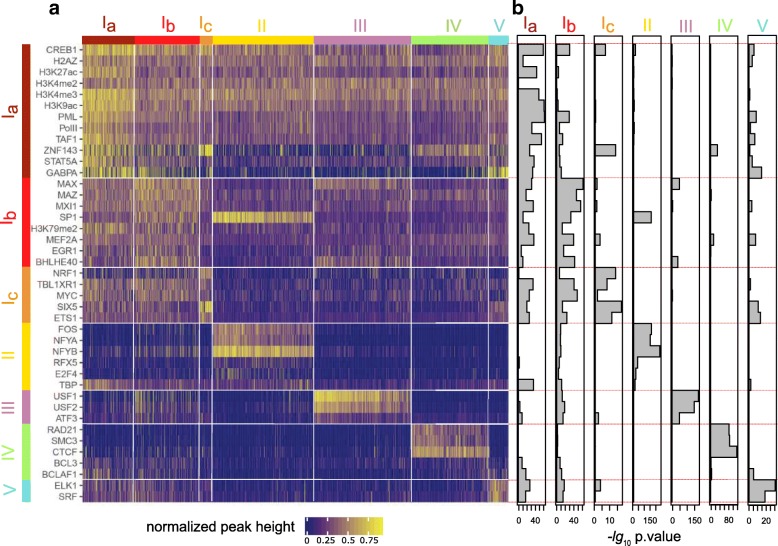
Visualization of the biclustering result for active promoters in GM12878 cell-line **a** ChIP-seq tracks (rows) and promoters (columns) are ordered according to the biclustering and displayed as a heatmap. The heatmap color corresponds to normalized peak height (see the “Methods” section) **b** Result of *t* test measuring cluster association for each row. The bars extend to the right to a height of the negative logarithm base 10 of the *p* value. A high *t* test value for a row and a certain cluster indicates that this row’s HM/TF is enriched in the respective cluster

We implemented a robust biclustering procedure (described in the “Methods” section) in order to cluster the columns and rows of a matrix in such a way that blocks of promoters co-occupied by a group of TFs or HMs become visible. Not the entire data matrix can be structured in this way and the algorithm yields a result only in as far as this is meaningful. For example, the matrix of promoter activity in GM12878 contains 6030 columns (promoters) and 60 rows (ChIP-seq experiments) altogether. From this data-set, the biclustering algorithm computed a structured heatmap with 1957 columns and 41 rows which is shown in the upper left of the complete matrix given in Additional file 1: Figures S1A zoom into the indicated structured submatrix for active promoters in GM12878 is shown in Fig. 1. The subsequent analysis will focus on this figure, while the analogous and very similar matrices for promoters active in K562 are in the Additional file 1: Figure S2).

**Fig. 2.**
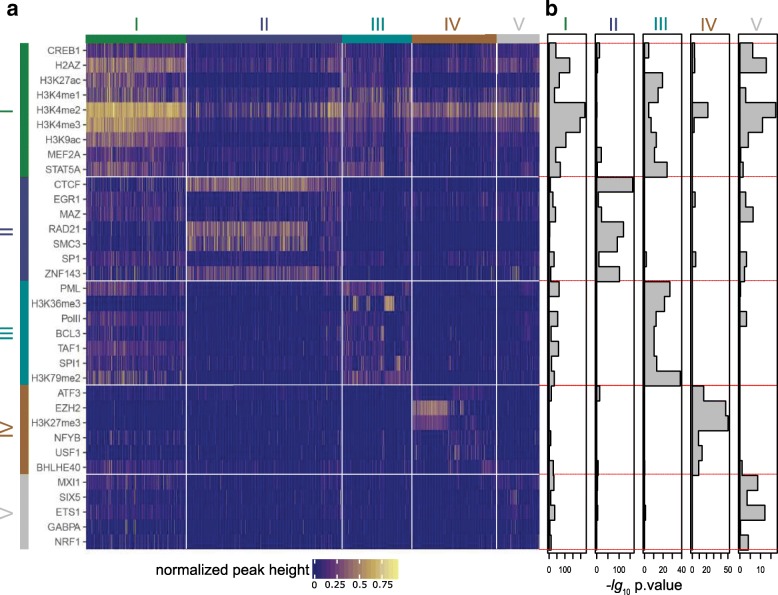
Visualization of the biclustering result for inactive promoters in GM12878 cell-line. **a** ChIP-seq tracks (rows) and promoters (columns) are ordered according to the biclustering and displayed as a heatmap. The heatmap color corresponds to normalized peak height (see the “Methods” section). **b** Result of *t* test measuring cluster association for each row. The bars extend to the right to a height of the negative logarithm base 10 of the *p* value. A high *t*-test value for a row and a certain cluster indicates that this row’s HM/TF is enriched in the respective cluster

The same procedure was applied to inactive promoters such that altogether four heatmaps were computed: For each of the two cell-lines, one heatmap for active promoters and one for inactive promoters. The structured submatrix of the inactive promoters in GM12878 is shown Fig. 2, while the analogous figure for K562 is in Additional file 1: Figure S3. For both active and inactive promoters, the clusters of columns (promoters) are color-coded at the top of the figure, while the row (ChIP-seq experiment) clusters are delineated by thin lines.

Besides using an algorithm that is robust in the sense that it does not report results that might be due to particular parameter settings (see the “Methods” section), two further precautions were taken against possible over-interpretation of computational results. First, an alternative biclustering algorithm based on a very different algorithmic paradigm was employed and found to yield highly similar results (see the “Methods” section and Additional file 1). Second, based on the reported clusters of promoters, we further test the association of individual rows with respect to the column clusters using a *t* test obtaining a measure how well a row fits into a bicluster. The resulting significance, measured as negative logarithm of the *t* test *p* value, is plotted towards the right adjacent to the matrix and grouped by the clusters to which membership is tested. Thus, each bar aligns to its respective row in the matrix, extending to the right and providing evidence in how far this row belongs to the respective cluster. As an example, consider the TF SP1 in Fig. 1 which associates also with promoters in cluster II, although the main affiliation of this TF is in cluster I. This way of associating a probability to the association of a row with a cluster further qualifies the information from the biclustering in an effort to prevent over-interpretation.

Similar biclustering results were obtained for promoters identified from CAGE peaks rather than RefSeq promoters. The corresponding heatmaps are shown in Additional file 1: Figures S4 and display the same division into a structured submatrix and an unstructured part. Note that the number of inactive TSSs in this CAGE-based definition is much larger than in the RefSeq-based definition, because whenever a CAGE cluster was observed in some other cell line, its absence in K562 or GM12878 is interpreted as an inactive TSS.

### Classes of active promoters

Based on occupancy patterns depicted by blocks in the heatmap, we have identified five groups of active promoters in GM12878 cell-line. Additional file 1: Figure S5 shows a bar plot of number of promoters in each of the cluster in both of the cell lines. We proceed to introduce these clusters based on the heatmap.

In the heatmap for the active promoters (Fig. 1), the activity-related histone marks H3K4me3, H3K27ac, and H3K9ac are clearly visible across the top rows, together with the rows for PolII and TAF. TATA-binding-protein (TBP) was sorted into another cluster, although visually TBP could be easily joined to these activity related marks. The *t* test supports this view in that testing TBP for cluster Ia versus the rest shows high significance. In GM12878, we named the first three clusters Ia, Ib, and Ic to indicate that visually they might easily be merged into one cluster. This cluster I further comprises a large group of transcription factors including, e.g., CREB, MYC, ETS1, and others. We observed a similar pattern for K562, where TBP is included in this activity-related cluster I, too.

**Fig. 3.**
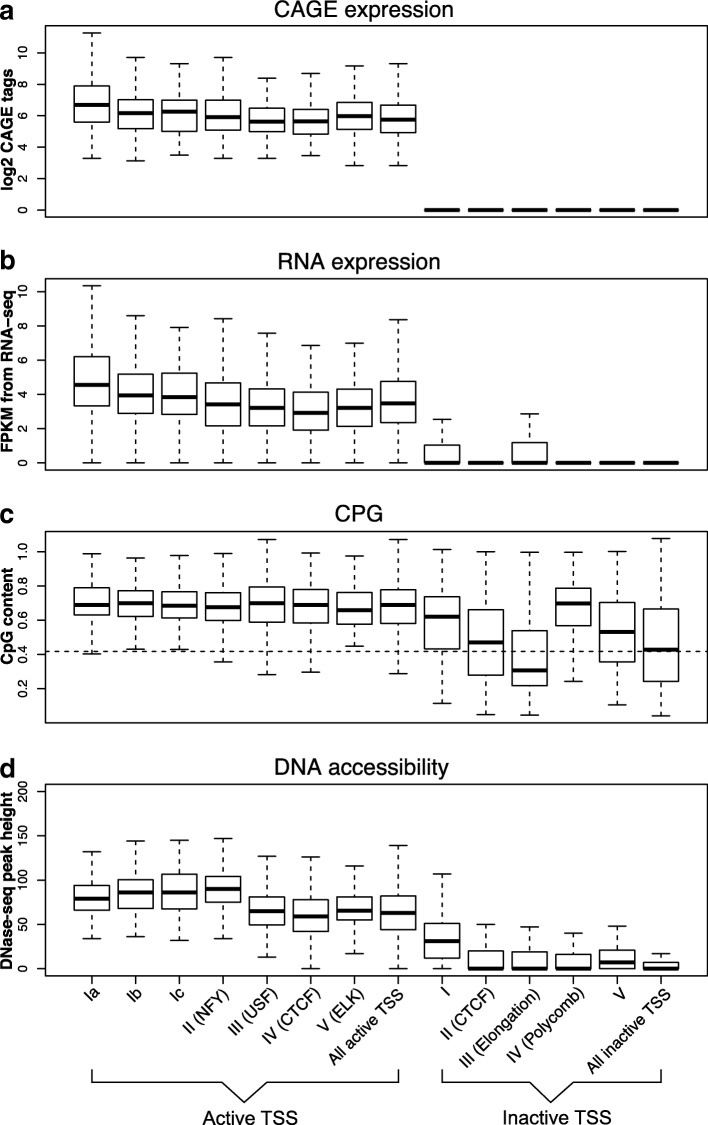
Expression measures and possible covariates associated to the individual clusters in GM12878 cell line box plots of values of **a** logarithm of number of CAGE tags. This is the criterion that served to determine active and silent promoters. **b** FPKM of RNA-seq for the target genes of the promoters in the cluster. **c** CpG contents in promoter region. The dashed line is the cutoff for defining high/low CpG contents human promoter. **d** Height of DNase accessibility peak in the promoters for each cluster. Cluster identifiers given at the bottom of the figure refer to all parts of the figure

Subdividing cluster I emphasizes some differences in the binding preferences of the transcription factors. In cluster Ia, several of the activity-related histone modification signals appear to be particularly high. This goes along with high activity of these promoters in terms of number of CAGE tags (Fig. 3a for GM12878 and Additional file 1: Figure S6 for K562). Cluster Ib is delineated especially by some of the basic helix-loop-helix (bHLH) transcription factors, although these also bind to the promoters of cluster III discussed below. Cluster Ic is characterized by promoters bound by SIX5, ETS1, and NRF1. Although included in Ia, ZNF143 binds to those promoters, too, as indicated by the *t* test.

Cluster II promoters (hereafter called “NF-Y-cluster”) are characterized by binding of NFYA and/or NFYB. The NF-Y transcription factors bind to the CAAT-box, a classical promoter motif [23]. It is clearly visible and supported by the *t* test, which the NF-Y-cluster includes FOS, as observed earlier by Struhl [24]. The *t* test also links SP1 to the NF-Y-cluster, although SP1 is visible both on cluster Ib and NF-Y-cluster promoters. In GM12878, SP1 has been assigned to cluster Ib by the algorithm, while in K562 data SP1 is assigned to the NF-Y cluster. Although cooperative binding of NF-Y and FOS, as well as NF-Y and SP1, have been reported in other studies [25], our analysis indicates a three-way synergy. For additional aspects including the corresponding sequence motifs see section “TF combinatorics in USF and NF-Y clusters”. Promoters in the NF-Y cluster show the standard activity-related marks but are notably devoid of signal from the other studied transcription factors.

In K562 cells, the algorithm groups together NF-Y, SP1, and FOS, too, and the *t* test supports this clustering (Additional file 1: Figure S2B). Additional file 1: Figures S7 and S8 shows the promoter occupancy with these three factors and demonstrates that many SP1 bound promoters indeed are also occupied by NF-Y and FOS. At the same time, SP1 binds to many more promoters, in line with observing SP1 occupancy also on cluster Ib promoters in the ChIP-seq biclustering matrix.

The USF family (USF1, USF2) transcription factors dominate cluster III (“USF cluster”). The USF binding motif is CACGTG [26], which is an instance of the classical E-box motif [27], known, e.g., from the regulation of clock genes [28]. Like many of the cluster I transcription factors, USF is also a bHLH factor. Interestingly, while there are reports of cooperative binding of USF and NF-Y in particular promoters [29, 30], we observe an anti-correlation in that promoters bind either the one or the other. This is further supported by the motif analysis which shows that the binding motifs of USF (E-box) and NF-Y (CAAT-box) tend not to co-occur in the same promoter (see section “Sequence motifs and enrichment analysis” and Additional file 1: Figures S9 and S10). Typical basic helix-loop-helix transcription factors like MAX and BHLHE40 also bind to the USF promoters, which hints at different dimers binding to the E-box. Although in K562 the biclustering algorithm did not assign USF2, the transcription factors USF1, USF2 and ATF3 are clustered together in GM12878, with MYC joined to that cluster in K562. MAX and BHLHE40 are also associated to this cluster by the *t* test.

Cluster IV (in K562 Cluster V, “CTCF cluster”) is dominated by CTCF/cohesin comprising CTCF itself together with cohesin components RAD21 and SMC3. Again, the group of promoters displaying this binding signal is largely devoid of binding of either NF-Y or USF. While CTCF is not considered a transcription factor, these promoters are at the same time occupied by Pol II, TBP, and TAF1. K562 also shows clustering of CTCF and SMC3, together delineating a particular albeit smaller group of promoters. The structural implications of this observation will be discussed further below.

The lower right corner shows another small cluster (cluster V in GM12878, cluster IV in K562) that is mainly characterized by ELK1 (binding site consensus CCGGAAGT) and SRF binding. These two proteins are known to interact and bind DNA jointly [31]. The *t* test analysis indicates that from the other clusters GABPA, SIX5, and ETS1 show similar binding behavior as ELK1 and SRF. ELK1 and GABPA are both members of the ETS transcription factor family [32].

Similar results for promoters identified from CAGE peaks only are shown in Additional file 1: Figures S11A (GM12878) and S11C (K562). Clusters are numbered as they are output by the algorithm. Clusters I, II, and III in S11A correspond to Ia-Ic from before comprising largely the same TFs and histone modifications. The NF-Y cluster from before corresponds to CAGE-based cluster IV. The RefSeq-based CTCF cluster from before becomes cluster VI in S11A. Only the USF cluster does not have a direct counterpart in the CAGE-based clustering, whereas the RefSeq-based SRF-elk cluster (V) gets combined with two additional TFs and is denoted V in S11A. For the active K562 TSSs in S23C, the correspondence between the biclusterings is comparable.

The two cell-lines under study, K562 and GM12878, were chosen based on the availability of many TF ChIP-seq experiments. While reproducing the entire analysis in another cell-line is therefore difficult, we did test for the existence of the proposed NFY, USF, and CTCF clusters in HeLa cells. ENCODE data for HeLa comprise ChIP-seq experiments for NFYA, NFYB, USF2, and CTCF, such that we could test our hypothesis there. Additional file 1: Figure S12 shows the promoter coverage patterns by these factors in HeLa cells after applying k-means (*k*=4) clustering to the data. This analysis confirms the existence of the NFY, USF, and CTCF clusters. Increasing the cluster number beyond 4 did not lead to new patterns. We also validated these clusters based on the CAGE-based TSS definition by showing their coverage pattern in GM12878 (Additional file 1: Figure S13) and K562 (Additional file 1: Figure S14). In both cases, the groups are again visible in the k-means sorting of the coverage patterns. Even the USF cluster on CAGE-TSSs becomes visible here, although it was not found in the CAGE-based biclustering.

### Classes of inactive promoters

In search for patterns associated with silent promoters we also applied the same data analysis strategy to those promoters which showed no CAGE tags (see the “Methods” section). Like for the active promoters, in the complete biclustering matrix for promoters inactive in GM12878 (Additional file 1: Figure S1), we home in on the rows and columns that are assigned to clusters (Fig. 2). The analogous matrix for K562 can be found in the (Additional file 1: Figures S1 and S3). The *t* test showing the membership of rows in a particular cluster is again printed to the right.

The first mechanism of repression that one would look for is by polycomb combined with H3K27me3. This can be found as part of cluster IV (cluster III in K562), where EZH2 and H3K27me3 mark around half of the promoters that are grouped into this cluster of around 400 promoters. These signals are hardly visible in any promoter outside this cluster.

Like among the active promoters, here too there is a cluster (cluster II) of promoters that is characterized by CTCF/cohesin. We inspected the RNA-seq data (Fig. 3b for GM12878 and Additional file 1: Figure S6B) and it shows that these promoters are clearly inactive. ZNF143 displays a very similar pattern, in line with the observation that it is a sequence specific chromatin-looping factor [33]. This is also supported by the K562 data. These observations will be discussed below in conjunction with the active CTCF-bound promoters.

There is a large cluster I that is characterized by H3K4me2, H3K4me3, and H2AZ. Yet, the other activity-related mark H3K27ac is weak in these promoters. The transcription-elongation-related mark H3K36me3 is mostly absent, while some of the promoters show H3K79me2. Given that the promoters for this particular analysis were selected to have almost no CAGE tags, these are contradictory signals. We are not looking at bivalent promoters because H3K27me3 is absent. The RNA-seq data shows extremely low signals for this cluster I Fig. 3b for GM12878 and Additional file 1: Figure S6B). However, the presence of H3K79me2 is an indicator of transcription, suggesting that we may be looking at unused alternative promoters. Indeed, for many of these promoters, we found an active alternative promoter further upstream (Additional file 1: Figure S15A). A similar explanation applies to cluster III (cluster IV in K562). Many promoters in this cluster display a combination of H3K36me3 and H3K79me2, again strongly indicating that in the cell type under study these genomic regions are part of a transcribed unit (Additional file 1: Figure S15B). Thus, in this cell type, transcription is not actually starting from these promoters but rather they are run over by transcription that had started elsewhere.

The clustering based on CAGE-defined TSSs lead to similar results. Additional file 1: Figures S11B and S11D show the two heatmaps for GM12878 and K562, respectively. The CTCF cluster (number II in Additional file 1: Figures S11B and S11D) and the polycomb-associated cluster (number IV in Additional file 1: Figure S11B and V in Additional file 1: Figure S11D) are clearly identifiable. There also exist the analogs to former cluster I (some active marks) and the H3K36me3 cluster (number V and III, respectively). The plots appear less succinct than for the RefSeq-based promoters due to the very large number of inactive CAGE-based TSSs.

### Differences in promoter occupancy patterns between cell-lines

**Fig. 4.**
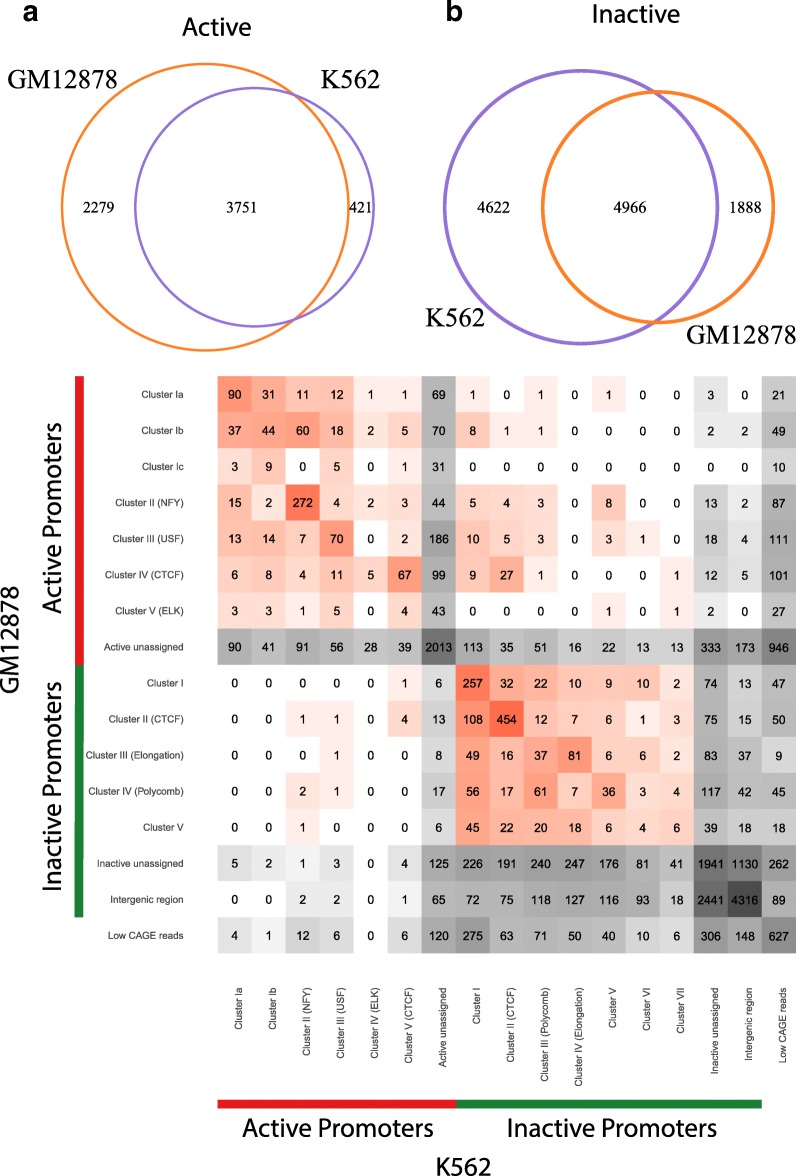
Comparison of promoter assignments between GM12878 and K562 cell-lines. **a**, **b** Overlap of active **a** and inactive **b** promoters between GM12878 and K562 cell lines shown as Venn diagrams. **c** Confusion matrix for overlapping TSS across different clusters in two cell lines. Entry in a matrix cell indicates the number of genes that belonging to that row (cluster) in the one cell line and that column (cluster) in the other cell line. “Active/inactive unassigned” TSS denotes ones that are not clustered by the biclustering algorithm (see the “Methods” section and Additional file 1: Figure S1). “Intergenic region” denotes TSSs without peaks in any ChIP-seq experiment (see the “Methods” section). “Low CAGE reads” denotes TSSs where the number of CAGE tags is positive but remains below the robust cutoff. Coloring reflects the counts in the matrix

The occupancy pattern and/or activity status of a gene’s promoter may differ between the two cell-lines that we have at hand for study. According to our criteria for active vs. inactive, 3751 genes are active in both cell-lines, while 4966 genes are inactive in both (Fig. 4a and b). Generally, fewer genes are categorized as active in K562 than in GM12878. Although comparison of the heatmaps for the two cell lines has shown similar clusters of active and inactive promoters, the actual genes making up the clusters differ. The confusion matrix of the shared genes across different clusters of the two cell-lines is shown in Fig. 4c, i.e., how many genes remain in the same cluster as opposed to switching between clusters. As an example, the row “Active – USF cluster” in GM12878 contains a cell with 70 genes which in the other cell-line are also in the USF cluster. On the other hand, the adjacent matrix cell indicates that 7 promoters from this GM12878 cluster show the occupancy pattern of the active NF-Y cluster in K562. One of these seven genes is Lmtk2, for which the promoter occupancy pattern is shown in Additional file 1: Figure S16: the promoter is occupied by USF in GM12878 and by NF-Y in K562. Further to the right in that row of the confusion matrix are the promoters that turn inactive in K562, e.g., three promoters which do so by moving to the (inactive) polycomb cluster. Among the promoters that are active in both cell lines, there is a tendency to stay within their clusters, while more of the inactive promoters change clusters. At the same time, one observes that the NSF-Y-cluster appears more stable than the USF cluster.

### Other covariates and functional categories

An interesting question concerns possible covariates associated to the individual clusters as well as possible functional characteristics. We have already discussed that genes in cluster I are generally more highly expressed than those from the other clusters. Figure 3a, b (Additional file 1: Figures S6A and B for K562 cell line) shows the levels of CAGE tags and RNA-seq expression levels across the clusters. It is apparent that the active promoters are targeting active genes and that cluster I is more active than the other clusters. The inactive promoters were chosen based on the lack of CAGE tags and accordingly also are very lowly or not at all expressed. We also find a strong enrichment of house-keeping genes [34] in this cluster (*χ*^2^ test *p* value = 1.26e −05, Additional file 1: Figure S17).

Another important covariate that could be linked to expression and the clustering of promoters is the CpG content of a promoter. Indeed, the vast majority of the active promoters is CpG rich, as shown in Fig. 3c(Additional file 1: Figure S6C for K562 cell line). Among the inactive promoters, clusters vary with respect to the CpG content of their promoters, with only the polycomb-repressed promoters comprising mostly high-CpG promoters. This is in line with the general assumption that the polycomb complex PCG2 gets recruited to CpG islands [35].

The DNase accessibility of a promoter is another indicator of its activity. Figure 3d (Additional file 1: Figures S6D for K562 cell line) shows the distribution of accessibility among the cluster promoters. Among the active clusters, cluster I together with the NF-Y cluster display a higher accessibility than the remaining clusters. The DNase accessibility signal for the inactive cluster is generally low, with the somewhat ambiguous inactive cluster I slightly higher than the others, although still much lower than any of the active clusters.

To further understand which histone modifications of transcription factors contribute the most to the gene expression value, we use a linear model to regress CAGE reads on the normalized ChIP-seq signals [36]. Overall, the Pearson correlation coefficient of all ChIP-seq signals and expression is *r*_*all*=0.878 and 0.879 in both of the two cell lines, respectively. Following the feature selection procedure used in [36], we further test all the combinations of 4 ChIP-seq experiments (487635 combinations) and record their Pearson correlation between the model predictions and the CAGE tags. We define the “good sets” as those yielding a correlation better than 95% of the maximal achievable 0.878 (resp. 0.879) correlation and then apply a hypergeometric test to determine those variables that are highly represented among the good sets. We found that TAF1, POlII, and TBP are the top three epigenetic marks that contribute the most to the gene expression (Additional file 1: Figure S18). Among the histone modifications, the most significant ones are H3K27ac, H3K9ac, and H3K79me2.

In search for possible functional implications of the clusters, we systematically tested the target genes of the promoters in the clusters for Gene Ontology enrichment using DAVID [37]. The five most significant categories with a *p* value better than 10^−5^ are summarized in Additional file 1: Table S3, while Additional file 2: (full_GO.xlsx) reproduces the entire DAVID output. For cluster I, this analysis yielded as the most significant hit the category “translational elongation,” which is in line with the prominence of house-keeping genes in this cluster. For the NF-Y and USF clusters, several categories relate to transcriptional control. The NF-Y clusters for the two cell lines largely overlap in terms of the content and accordingly the GO enrichment results are similar. In contrast hereto, the USF clusters contain different genes in the two cell-lines and the enrichment results are accordingly dissimilar. From the presented statistics no clear conclusion as to functional implications of the promoter clusters can be made.

### Sequence motifs associated to clusters

A key question in our analysis of promoter clusters concerns the difference between TF occupancy and existence of TF binding motifs. We therefore investigated whether promoter clusters are supported by sequence motifs and whether there are further sequence motifs beyond the known ones for the ChIP-seq TFs. To this end, we conducted motif-search in promoter clusters using all motifs from the Jaspar database (Jaspar 2014) using the MAST tool from the MEME suite ([38], see the “Methods” section for details). This analysis recovered the binding motifs of the ChIP-seq transcription factors but no further motifs beyond that (data not shown). We conclude that the observed clustering is not a product of binding of further transcription factors, in as far as the binding motifs are contained in Jaspar.

We further asked whether in the ChIP-seq experiments the TFs bind all promoters containing that TF’s binding site, or whether they only bind a subset of available binding sites. To this end, we computed the motif hits for the binding sites in all the promoters and depict the resulting motif match scores as rows of a matrix whose columns are ordered like in the biclustering matrix (Additional file 1: Figures S9 and S10). Some motifs occur in many promoters and across all clusters, like, e.g., the SP1 motif. This is in stark contrast to the clearly defined subset of SP1-bound promoters according to the ChIP-seq heatmap (Fig. 1 and Additional file 1: Figure S2). On the other hand, the motif hits for the NF-Y binding CAAT-box and the USF binding motif, the E-box, are remarkably concentrated in their respective cluster of TF-bound promoters. This suggests that the existence of the respective motif in the promoter is in itself sufficient for TF binding and possible activation of that gene.

### TF combinatorics in USF and NF-Y clusters

**Fig. 5.**
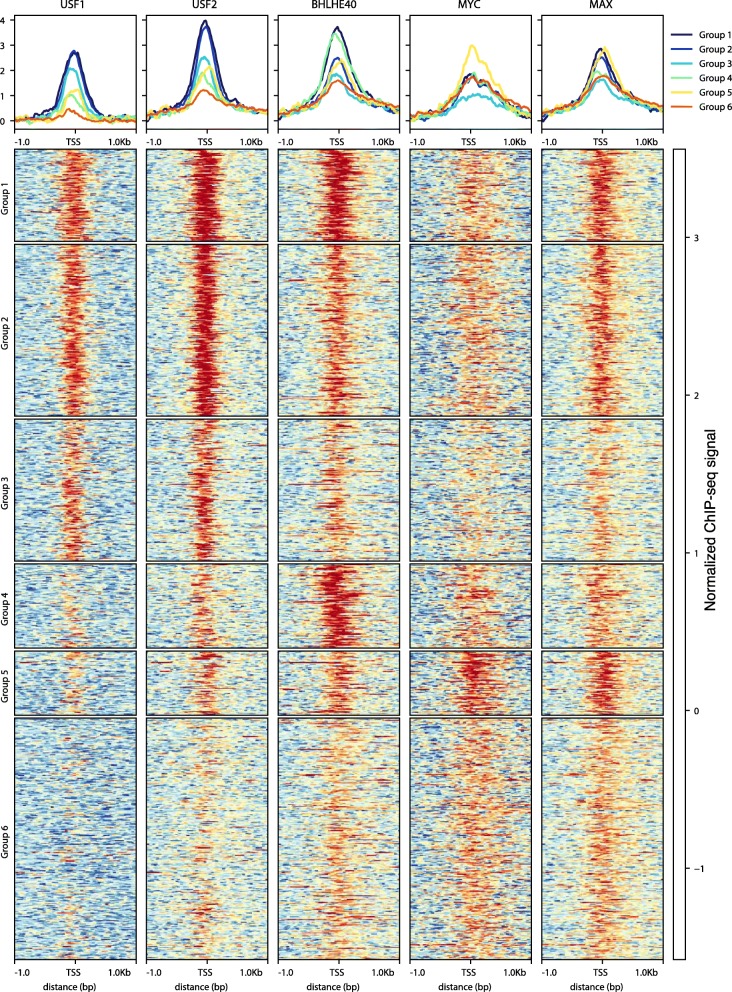
Binding combinatorics in E-box containing promoter density plots and coverage patterns for ChIP-seq signals of TFs recognizing the E-box (USF1/2, BHLHE40, MYC, MAX) in ±1 Kbp window of the selected TSSs in GM12878 cell line. Promoters are ordered in columns and grouped according to k-means clustering

As pointed out above, the hallmark of the USF cluster promoters is the E-box motif, which is shared by many bHLH proteins. To study possible combinatorial TF binding, we focus on five proteins from this family [39] available in our data-set: USF1/2, BHLHE40, MYC, and MAX. We use MAST (see the “Methods” section) to find promoters motif hits in a [ −200, 200] promoter window with a *p*-value cutoff 0.001. Discarding those promoters without ChIP-seq peaks for all five proteins, we are left with 880 promoters in both the GM12878 and K562 cell line. The normalized ChIP-seq read coverage pattern in the promoter region is shown in Fig. 5. (see also Additional file 1: Figure S19 for K562). The promoters (rows) are sorted by *k*-means clustering.

USF1 and USF2 most of the time bind together with BHLHE40 and MAX (Fig. 5*k*-means groups 1, 2, and 3, Additional file 1: Figure S19 *k*-means groups 2–4). MYC displays a strong ChIP-seq signal only when it binds together with MAX, apparently in the well-known MYC-MAX complex (Fig. 5*k*-means group 5, Additional file 1: Figure S19 *k*-means group 6). In GM12878, we observe a group of BHLHE40-specific promoters (Fig. 5*k*-means group 4) and K562 has a group of MAX-specific promoter (Additional file 1: Figure S19 *k*-means group 1). This appears to be the only promoter set that is different between the two cell lines. Thus, the similarity of TF occupancy pattern in different cell lines suggests a conservation of binding combinatorics across cell types and is consistent with observed importance of bases flanking the core E-box [40] for specific binding.

**Fig. 6.**
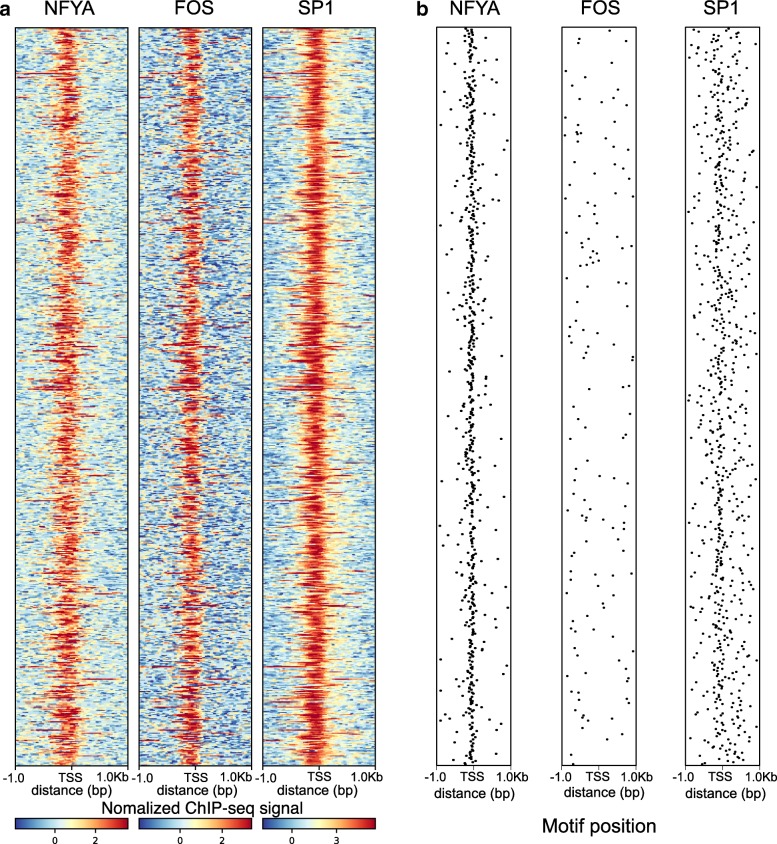
Binding patterns of NFYA, FOS, and SP1 compared to motif occurrence. **a** Coverage patterns for ChIP-seq signals of NFYA, FOS, and SP1 in a ±1Kbp window of selected TSSs in GM12878. **b** Estimated motif position for the same TFs and TSS windows as **a**. While motif locations for NFYA and SP1 are concordant with the ChIP-seq signal, the FOS motif locations cannot explain the FOS ChIP-seq signal

The NFY-cluster contains many promoters that, besides NF-Y, also bind FOS and SP1 (see Additional file 1: Figures S7 and S8. All of these TFs bind to different sequence motifs: While NF-Y binds to the CAAT element, SP1 binds to the GC-box (GGGCGG, [41]), and the reported FOS binding site consensus is TGACTCA [42]. Taken together, this leads to the question where the binding sites of the three TFs are located with respect to the motifs, to each other and to the TSS. To study this, we selected 711 active promoters based on their ChIP-seq peaks for NFYA, FOS, and SP1 (Fig. 6a). Figure 6b shows the motif position (if the motif exists in the promoter) in each TSS window according to MAST in the GM12878 cell line (Additional file 1: Figure S20 for K562 cell line). The NFYA motifs align almost precisely on the TSS, while the SP1 motif also aligns around the TSS, although with larger variation. However, in spite of the FOS ChIP-seq signal, only few promoters contain a FOS binding motif, and even if they do, the position of the predicted motif hit seems not to coincide with the location of the ChIP-seq peak, which lies close to the TSS. A possible explanation would be that, on these promoters, NFYA, FOS, and SP1 form a complex within which FOS does not directly interact with the DNA, or at least not through its normal binding motif. Such a complex might also be formed through chromosomal looping as recently suggested, e.g., for the interaction of glucocorticoid receptor and AP-1 [43].

### CTCF and spatial promoter-enhancer interaction

**Fig. 7.**
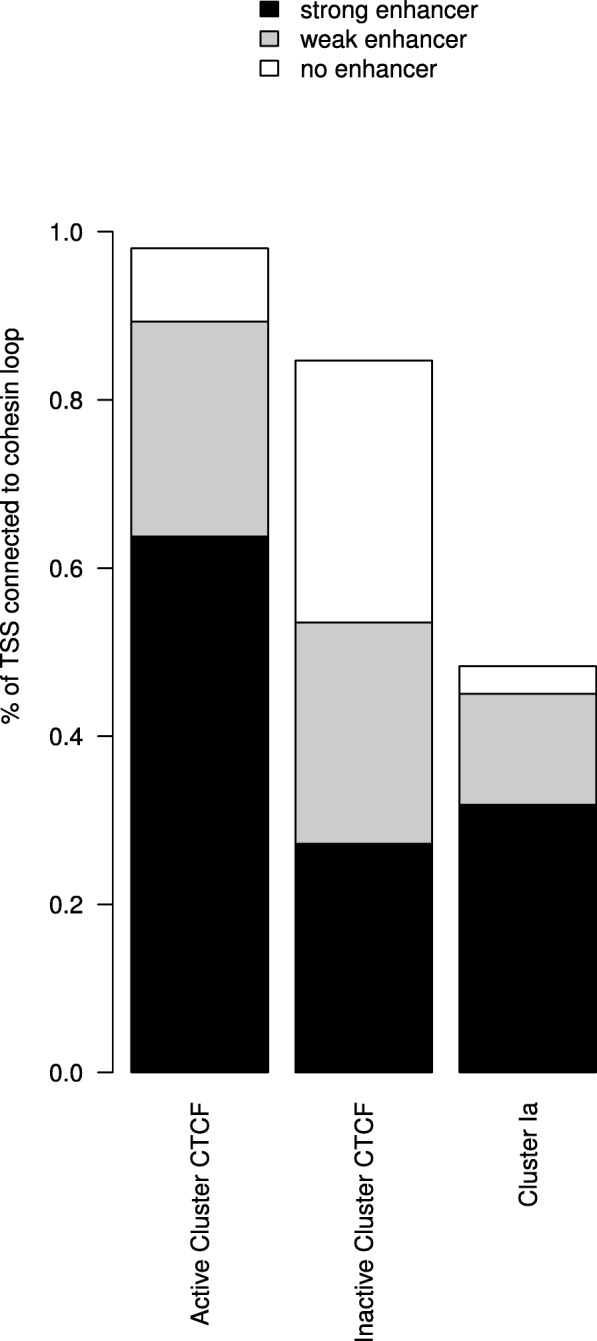
Number of cohesin loops and putative enhancers Barplot for proportion of TSSs that form cohesin loops in the active CTCF cluster, the inactive CTCF cluster and the active cluster Ia. Bars contain the proportion of remote interacting sites of TSSs, which overlap with strong enhancers (black), weak enhancers (gray) or no enhancers (white) based on ChromHMM-predicted enhancers. If remote sites of a TSS overlap with both strong enhancers and weak enhancers, it is shown in the Figure as a strong enhancer (black)

The fact that we find a distinct cluster of CTCF binding promoters that is characterized by the combined ChIP-seq signals of CTCF, RAD21, and SMC3, prompts the question whether CTCF/cohesin might mediate the interaction to enhancers which loop to these promoters. To answer this, we integrated a ChIA-PET data-set on spatial chromosomal interactions mediated by CTCF [44] into our analysis. In this data-set, one CTCF-bound promoter will typically show spatial interaction with several genomic regions. Almost all promoters from the active CTCF cluster (98.03%) and 84.67% of promoters from the inactive CTCF cluster show a spatial interaction. For comparison, in cluster Ia, which contains highly expressed genes, roughly only 48.35% the promoters interact spatially with some other genomic region according to the ChIA-PET data (Fig. 7).

The existence of spatial interactions suggests that the distal regions of these interactions might overlap with enhancers targeting the respective promoters. To test this, we downloaded from ENCODE the ChromHMM-generated enhancer annotation (GSM936082) [45] to compare to the distal ChIA-PET regions. For the purpose of counting overlapping regions, we extend the distal region by 500 bp at either side and check for overlap with a ChromHMM strong or weak enhancer. The respective figures are integrated into the bars in Fig. 7: 91.11% of promoters in the active CTCF cluster loop to at least one region predicted to be an enhancer, with 65.04% looping to what is predicted as a strong enhancer. Promoters from the inactive CTCF cluster loop to a strong predicted enhancer in 32.13% of cases.

**Fig. 8.**
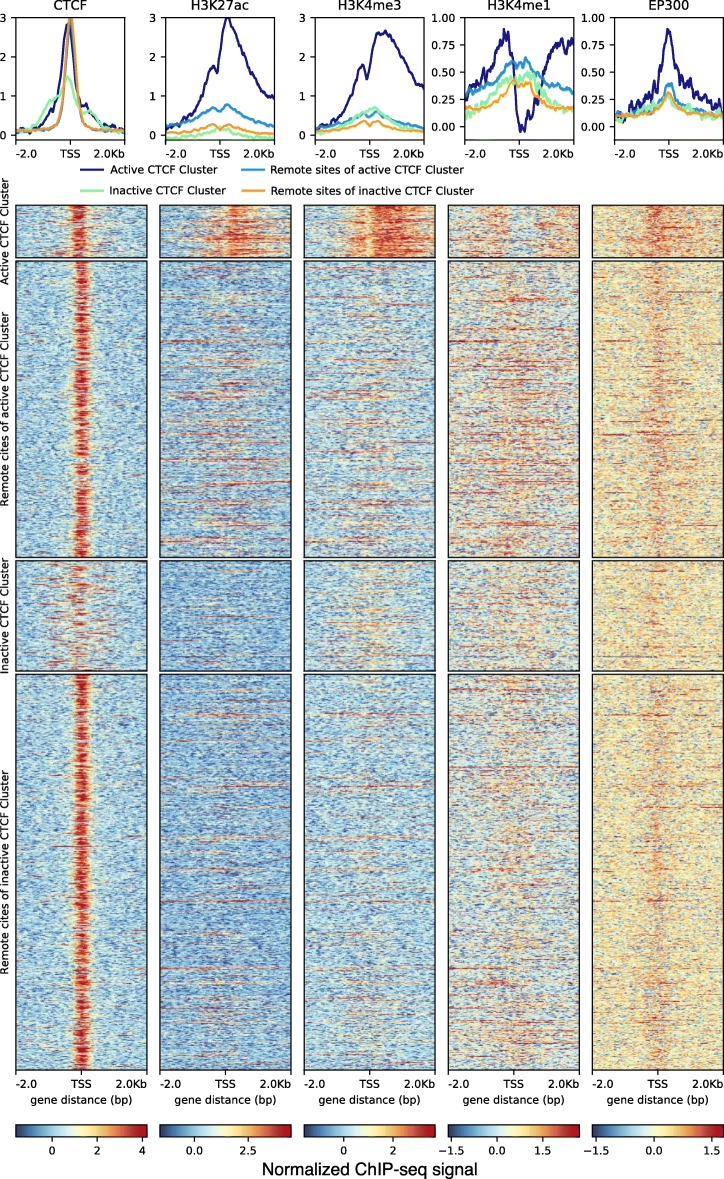
Histone modifications in promoters in active and inactive CTCF clusters shown together with their ChIA-PET-derived interacting regions The row “Active CTCF cluster” shows coverage patterns of the active promoters in the CTCF cluster. These are characterized by activity signals (H3K27ac, H3K4me3). The row “Interacting regions of active CTCF cluster” shows the coverage patterns for the ChIA-PET derived interacting regions, the putative enhancers. They display activity marks as well as the enhancer marks H3K4me1 and p300. The row “Inactive CTCF cluster” displays the promoters from the inactive CTCF cluster. These promoters lack the activity marks. Via CTCF/cohesin they link to interacting regions (bottom row), which likewise lack activity marks. The top of the figure shows density plots for the histone marks discussed. All data for GM12878 cell line

The difference between active and inactive CTCF clusters can be further explored by inspecting the histone modifications for the respective promoters and their interacting regions. Coverage patterns for CTCF, H3K27ac, H3K4me3, H3K4me1, and P300 for the active promoter regions (upper row), for their interacting regions (second row), for inactive promoters (third row) and their interacting regions (bottom row) are shown in Fig. 8. The promoter regions are centered on their TSS. While in the active promoters the CTCF signal also focuses on the TSS, this signal is dispersed in the inactive promoters. In the interacting regions—recall that there are more interacting regions than promoters due to the multiplicity in the ChIA-PET assignments—the regions were centered on the summit of the CTCF ChIP-seq peak which leads to the visual impression of a peak alignment. The H3K27ac mark is clearly visible for the active promoters and absent in the inactive promoters. Many of the regions interacting with active promoters also show the H3K27ac signal, confirming the ChromHMM designation as an enhancer. Likewise, H3K4me3 is strong on the active promoters, while their interacting regions rather show H3K4me1. H3K4me1 appears much weaker in inactive promoters. The acetylase P300, a major marker of enhancers [46], is clearly visible on the putative enhancers, as well as on the promoters

## Discussion

There has for a long time been a keen interest in determining principles of human promoter architecture. Since sequence motifs have not sufficed to achieve this, we turned to analyzing transcription factor occupancy in promoters as determined in the ENCODE project, in conjunction with sequence. We proceed in a top-down manner starting from the entirety of promoters with their ENCODE ChIP-seq signals and apply biclustering to delineate subgroups of promoters that share particular histone modifications and/or transcription factors. Our study fills a gap between sequence-based promoter studies (e.g., [5]) and the global occupancy analysis, like, e.g., in [14]. Other authors have studied the global regulatory network [47] or focused on NF-Y and its co-factors [6]. Our goal was the delineation of subsets of stereotypical human promoter architectures.

Among the active promoters, we identified the NF-Y binding promoters, containing the CAAT-box motif, as one distinct promoter cluster. Like other authors before us [24], we observe that NF-Y binding frequently goes along with binding of FOS and Sp1. While Sp1 binding sites occur ubiquitously, we have shown that this combination is highly characteristic of a subgroup of promoters.

Another subgroup is characterized by the basic helix-loop-helix factor USF binding to its binding site, the E-box. The NF-Y cluster and the USF cluster appear almost mutually exclusive, reinforcing the view that these two groups constitute two characteristic promoter architectures.

In the cell types studied, there is also a small cluster characterized by binding of the ETS TF. At the same time, there is a large group of promoters that apparently lack any characteristic TFs or TF-combinations, but clearly show the activating histone modifications. While the groups discussed are the result of a clearly visible clustering pattern, there are many more promoters for which—at least with the available transcription factor ChIP-seq experiments, no cluster structure is visible. We conclude that certain characteristic promoter types exist, but only a part of all promoters falls into any of these subgroups.

A further promoter cluster is characterized by binding of CTCF in the promoter region. For this group, we presented evidence suggesting that CTCF and cohesin are responsible for the interaction between the bound promoter and one or more enhancers. This putative arrangement differs from the one where, within a TAD (topologically associated domain [48, 49]), promoters and enhancers can cluster in space without direct involvement of CTCF/cohesin. It remains to be seen whether the two arrangements have different functional consequences.

On the side of the inactive promoters, we were surprised to see that the number of promoters repressed by polycomb is fairly small, thus raising the question whether there are other “mechanisms of repression,” or whether repression is just the absence of activation. We also had expected to see a stronger role for the repressive mark H3K9me3. However, the signal for this mark is low across all experiments (see Additional file 1: Figure S1) and it is unclear whether its absence actually constitutes a biological signal or is due to some technical issues.

Other authors [6, 16] investigated the question whether genes with promoters bound by NF-Y belong to particular functional classes of genes as given, e.g., in the Gene Ontology database. We also tested our derived promoter clusters for such target categories but the results remain rather generic and unconvincing. Although each study, including our own, finds some enriched GO term, those functions are either not consistent within a cluster or they are so generic that they are hardly informative. Thus, we suspect that TF combinatorics might actually lack a systematic link to functional categories.

## Conclusion

In summary, basing this analysis on a combination of TF occupancy and motif analysis, we have defined stereotypic patterns delineating a novel grouping of promoters. This grouping opens up interesting new questions concerning the transcription factor complexes at the respective promoter groups, as well as questions on the evolutionary origin of these groups.

## Methods

### Data-sets and preprocessing

#### Potential transcription start sites

We downloaded coordinates of potential transcription start sites (TSSs) listed in the RefSeq annotation (hg19, release from December 10, 2014, from UCSC database). Cases where two or more TSSs lie within a 1 Kbp window are ignored altogether. This leaves 22164 annotated TSSs.

#### Cell type-specific activity status of TSS

TSS activity information is obtained from the CAGE (Cap Analysis of Gene Expression) analysis in the FANTOM 5 project [20]. CAGE measures RNA expression level by capturing and sequencing the initial 20 nucleotides from the 5 ^′^ end of an RNA. We downloaded CAGE data for the GM12878 and K562 cell lines [50]. CAGE peaks are called following the method of FANTOM consortium (decomposition peak identification (DPI) [51]). TSSs are divided into active and inactive ones. We define a TSS which has robust peaks in its [−500,+500] window around the TSS as active, whereas an inactive TSS is defined as not even having a FANTOM-defined permissive peak in that window. This process yields 6030 active and 14390 inactive TSSs in GM12878 and 4173 active and 15494 inactive TSSs in K562 cell line. We further discard inactive TSSs when located in an intergenic region. In the end, we are left with 6854 inactive genes in GM12878 and 9588 inactive promoters in K562.

We also download RNA-seq from ENCODE/Caltech (GSM958730,GSM958731). We apply cufflinks [52] with default parameters using RefSeq annotation described before on all the replicates. FPKM values of each TSS are used.

#### ENCODE ChIP-seq data

We downloaded ChIP-seq data and DNase-seq data (GSM736496/GSM736629) for the GM12878 and K562 cell lines from the ENCODE project [10]. This data-set comprises ChIP-seq data for 11 histone modifications and 49 TFs or chromatin-associated proteins. A detailed list is given in Additional file 1: Table S1 and S2. After merging the replicate bam files for each ChIP-seq and DNase-seq experiment, we use MACS [53] to call peaks with default parameter and the “–call-subpeaks” option. Then, for each TSS the height of the peak closest to the TSS from within a [−1000,+1000] bp window around it is recorded. This value we normalize within each ChIP-seq experiment by linearly mapping the peak heights to the interval [0,1]. We reduce the influence of outliers by holding out the top 0.05*%* of values before the linear transformation and subsequently setting these values to 1. For depicting the ChIP signals in the promoter regions as coverage patterns, we use deeptools [54] and normalize the ChIP-seq signal against its input using the “bamCompare” function under default parameters.

To validate our observation in GM12878/K562 cell line, we download four ChIP-seq (NFYA, NFYB, CTCF, and USF2) together with their Input in HeLa from ENCODE (see Additional file 1: Table S1 and S2).

#### CTCF and ChIA-PET data

We downloaded the genomic coordinates of interacting genomic regions as determined by ChIA-PET for CTCF in GM12878 [44] (GSM1872886). For each promoter in the CTCF cluster in active and inactive biclustering results, we looked for CTCF loops which have one side mapped to these promoter regions, and record the genomic loci for the other side of the loop (we call it the “interacting region”). If more than one loop is found interacting with a promoter region, the loop with the highest H3K27ac signal at the remote CTCF binding region is chosen. Since the remote CTCF binding regions differ in size, we choose the site which has the highest CTCF ChIP-seq read coverage as the center of the region and extend ±1 Kbp for depiction of the coverage patterns.

### Transcription factor binding motifs

As a resource for TF binding motifs, we used the Jaspar database [26]. To calculate the motif match score, we use the program MAST [38] from the MEME suite [55], using its default parameters and reporting all hits. If there is more than one hit in a promoter region, we take the hit with the smallest *p* value for further analysis.

### Gene ontology enrichment analysis

We use DAVID functional annotation tool (version 6.7) [37] to do gene ontology enrichment analysis. For enrichment analysis on each active/inactive clusters, we use all active/inactive genes in the cell line as the background, for both of the cell lines. We present here enriched GOTerm in three categories: biological process (BP), cellular component (CC), and molecular function (MF). In Additional file 2, we listed all the GO terms that with adjusted *p* value cutoff =0.05 and fold enrichment cutoff =2 for all clusters and both cell lines.

### Sorting the heatmaps by biclustering

Our method for clustering the rows and columns of the CHIP-seq data matrix into a visually understandable heatmap is based on a robust version of the s4vd biclustering algorithm [56], which is available as an R package. s4vd solves the biclustering problem with a procedure that is based on the singular value decomposition. The result of biclustering a matrix is a coupled set of column and row clusters, where these cluster pairs are visible as sub-rectangles of the matrix with rows and columns permuted correspondingly. We use the default parameter settings of s4vd.

Due to its use of a randomized selection step, s4vd produces somewhat different results in different runs of the program. We exploit this with the goal of obtaining robust biclustering results by extracting the common cluster assignments from many runs. To this end, we run s4vd many times and determine the solution with the largest number of column clusters (a tie gets broken randomly). We call this the target clustering. Then, for each of the other solutions obtained from the other runs of s4vd, we determine an optimal assignment of its clusters to the target clustering. This is done by solving a linear assignment problem on the confusion matrix of the two cluster systems. The linear assignment problem is solved using lp.solve from R [57]. Once all the clusters from all solutions are assigned to the target clustering, we determine the frequency at which a column gets mapped into a target cluster. Finally, we only keep columns for which there exists a target cluster to which it gets mapped with a frequency of more than 0.5, i.e., more than half the solutions would clusters this column consistently into a similar looking target cluster (by virtue of the optimal assignment).

In a biclustering result, there exists a connection between a column and a row cluster. Thus, once the column clusters are fixed by the above procedure, one can also determine how often a particular row gets associated to a row cluster. Again, we keep those rows, which are associated to one cluster in more than half of the solutions, and assign this row accordingly. This also leads to the *t* test *p* values reported next to our heatmaps: For one row, the test compares the mean of the values among the entries in the associated column cluster with the mean of the values outside the column cluster. This visualization is a further precaution against over-interpretation of the biclustering results. We uploaded our original data matrix and source code on the github [58].

To further ensure that the computational results which we interpret are not due to the specifics of our algorithm, we applied a second biclustering algorithm which is based on a very different computational principle. Rather than relying on the SVD, we first use *k*-means clustering on the TSSs (columns) of the ChIP-seq matrix. Additional file 1: Figure S21 shows how the value of *k* was selected. To then determine the association of a row with a cluster, we test each row with a *t* test. Like in the visualization procedure described above, the *t* test measures in how far a particular column cluster divides the row of active promotes into two different regimes of high and low values, respectively. For a given column cluster, the rows with a *t* test *p* value better than 0.001 form the row cluster associated to that column cluster. Both biclustering procedures are depicted graphically in Additional file 1: Figure S22. Additional file 1: Figure S23 shows the confusion matrices between the biclustering resulting from the two algorithms. It is apparent that the clusters that we have interpreted are stably reproduced also by the *k*-means *t* test-based algorithm.

### Promoter definition based on CAGE tags

To not rely solely on the RefSeq promoter annotation, we also alternatively use the same CAGE tag data as above to define promoter location.

Here, we describe how promoters were derived from FANTOM 5 CAGE data. The FANTOM consortium has provided a CAGE-tag based TSS annotation which we downloaded from their website [50] Here, we also choose TSSs under the robust cutoff. There are 217572 TSSs in this annotation. Note that this annotation comprises CAGE tags from all the cell lines studied in FANTOM 5. In particular, it includes all possible isoforms and a gene might have several possible TSSs in a very short region, see example Figure in Additional file 1: Figure S24.

In this example, we have 6 TSS for Nat10 within an interval of 1000 bases. Although the annotation might be very accurate (i.e., to base pair resolution), it is not helpful to analyze all these TSS individually, because the resolution of the ChIP-seq data with which we annotate the promoters is much lower. Thus, we group CAGE TSS into potential promoter regions before we apply our biclustering method. We first join together TSSs with a distance shorter than 200 bp into a TSS cluster. We then exclude TSS clusters which extend over a length of more than 1000 bps, or where there are CAGE TSSs on both plus and minus strand in the same cluster, or when there are TSSs from more than one annotated gene in a cluster, see illustration in Additional file 1: Figure S25. After this step, we have 43675 TSS clusters with 121129 TSSs.

In order to assign an activity status to such a potential promoter, we extend 500 bps for each TSS cluster region and search for robust or permissive CAGE peaks in the K562/GM12878 cell lines. If there is one or more robust peaks of a cell line in one TSS region, then the TSS cluster is deemed active in this cell line. If there is not even a permissive peak in this TSS cluster region, then the TSS cluster is deemed inactive. Note that this step is strand specific.

After this step, we have 6684 active TSS clusters comprising 22340 TSSs and 31969 inactive TSS clusters comprising 82957 TSSs in the GM12878 cell line. For K562, we have 4375 active TSS clusters (13951 TSSs) and 35649 inactive TSS clusters (94654 TSSs). To save the expense of computing, we exclude inactive TSSs without any ChIP-seq signals in the ±1 Kbp TSS windows from the analysis. In addition, since two very close TSSs usually have the same ChIP-seq signals in their TSS windows, we keep only one row in the input matrix if two or more TSSs in a TSS cluster have exactly the same values of all ChIP-seqs.

The input matrix of active/inactive TSS of GM12878 for biclustering contains 19316/30634 TSSs, the input matrix of active/inactive TSS of K562 for biclustering contains 12861/41013 TSSs.

## Additional files

Additional file 1 Supplementary tables and figures. Additional Table S1–S3. TableS1: Downloaded data from ENCODE (GM12878/K562). TableS2 : Details of downloaded data control (GM12878/K562). TableS3: Top five significant GO categories for the active cluster in both cell lines. Additional Figures S1–S25. FigS1: TSSs assigned to clusters according to biclustering algorithm. FigS2: Biclustering result of active TSS in K562 cell-line. FigS3: Biclustering result of inactive TSS in K562 cell-line. FigS4: TSSs assigned to clusters according to biclustering algorithm based on CAGE tags. FigS5: Proportion of each cluster among all assigned promoters. FigS6: Expression measures and possible covariates associated to individual clusters in K562 cell line. FigS7: NFY co-binding pattern in GM12878 cell line. FigS8: NFY co-binding pattern in K562 cell line. FigS9: TF motif hits in promoters in GM12878 cell line. FigS10: TF motif hits in promoters in K562 cell line. FigS11: Biclustering results based on CAGE tags. FigS12: Validation of NFY, USF, and CTCF clusters in HeLa cells. FigS13: Validation of NFY, USF, and CTCF clusters in GM12878 cells based on CAGE tags. FigS14: Validation of NFY, USF, and CTCF clusters in K562 cells based on CAGE tags. FigS15: Examples of inactive TSS embedded in an active gene. FigS16: Example of promoter bound either by NFY or USF in the two cell lines. FigS17: Transcript type and function analysis for genes in each cluster. FigS18: Histone modifications and transcription factors significantly contributing to gene expression. FigS19: Binding combinatorics in E-box containing promoters in K562 cell line. FigS20: Binding patterns of NFYA, FOS and SP1 compared to motif occurrence in K562 cell line. FigS21: Sum of square errors and coefficient in *k*-means clustering under different number of clusters, for active/inactive TSSs for GM12878/K562 cell line. FigS22: Biclustering methods. FigS23: Comparison of biclustering and *k*-means methods. FigS24: Example of a promoter whit multiple CAGE tag annotations. FigS25: Overview of promoters definition based on CAGE tags. (PDF 11,502 kb)

Additional file 2 Entire DAVID output with adjusted p value cutoff =0.05 and fold enrichment cutoff =2. (XLSX 92 kb)
