# Supplementary material for: Classifying human promoters by occupancy patterns identifies recurring sequence elements, combinatorial binding, and spatial interactions
Source: BMC Biol. 2018 Nov 15;16:138. doi: 10.1186/s12915-018-0585-5 (PMC6238301; doi:10.1186/s12915-018-0585-5)
Supplement: Supplementary file 1 — Supplementary tables and figures. Additional Table S1–S3. TableS1: Downloaded data from ENCODE (GM12878/K562). TableS2 : Details of downloaded data control (GM12878/K562). TableS3: Top five significant GO categories for the active cluster in both cell lines. Additional Figures S1–S25. FigS1: TSSs assigned to clusters according to biclustering algorithm. FigS2: Biclustering result of active TSS in K562 cell-line. FigS3: Biclustering result of inactive TSS in K562 cell-line. FigS4: TSSs assigned to clusters according to biclustering algorithm based on CAGE tags. FigS5: Proportion of each cluster among all assigned promoters. FigS6: Expression measures and possible covariates associated to individual clusters in K562 cell line. FigS7: NFY co-binding pattern in GM12878 cell line. FigS8: NFY co-binding pattern in K562 cell line. FigS9: TF motif hits in promoters in GM12878 cell line. FigS10: TF motif hits in promoters in K562 cell line. FigS11: Biclustering results based on CAGE tags. FigS12: Validation of NFY, USF, and CTCF clusters in HeLa cells. FigS13: Validation of NFY, USF, and CTCF clusters in GM12878 cells based on CAGE tags. FigS14: Validation of NFY, USF, and CTCF clusters in K562 cells based on CAGE tags. FigS15: Examples of inactive TSS embedded in an active gene. FigS16: Example of promoter bound either by NFY or USF in the two cell lines. FigS17: Transcript type and function analysis for genes in each cluster. FigS18: Histone modifications and transcription factors significantly contributing to gene expression. FigS19: Binding combinatorics in E-box containing promoters in K562 cell line. FigS20: Binding patterns of NFYA, FOS and SP1 compared to motif occurrence in K562 cell line. FigS21: Sum of square errors and coefficient in k-means clustering under different number of clusters, for active/inactive TSSs for GM12878/K562 cell line. FigS22: Biclustering methods. FigS23: Comparison of biclustering and k-means methods. FigS24: Example of a promoter whit mult [file 12915_2018_585_MOESM1_ESM.pdf]

Grouping of human promoters by occupancy patterns yields recurring sequence elements, binding combinatorics, and spatial interactions
Additional file 1

Xinyi Yang and Martin Vingron

Supplementary Tables

Table S1: Details of downloaded data from ENCODE (GM12878/K562)

Histone Modifications					
Name	Lab	Replicates	Antibody	Control	GEO Sample Accession
H2AZ		2	07-594		GSM733767/GSM733786
H3K27ac		2	ab4729		GSM733755/GSM733656
H3K27me3		3/2	07-449		GSM733758/GSM733658
H3K36me3		2	ab9050		GSM733679/GSM733714
H3K4me1		2	ab8895		GSM733772/GSM733692
H3K4me2	Broad	2	ab7766	Std	GSM733769/GSM733651
H3K4me3		2	07-473		GSM733708/GSM733680
H3K79me2		2	39143		GSM733736/GSM733653
H3K9ac		2	ab4441		GSM733677/GSM733778
H3K9me3		3/2	ab8898		GSM733664/GSM73377
H4K20me1		2	ab9051		GSM733642/GSM733675

Proteins					
Name	Lab	Replicates	Antibody	Control or Protocol	GEO Sample Accession
ATF3	HAIB	2	sc-188	PCR 1-round/biorupter	GSM803508/GSM803380
BCL3	HAIB	2	sc-185	biorupter/PCR 1-round	GSM803342/GSM803518
BCLAF1	HAIB	2	sc-101388	biorupter/PCR 1-round	GSM803509/GSM803515
BHLHE40	SYDH	2	NB100-1800	IgG-mus/IgG-rab	GSM935430/GSM935616
CEBPB	HAIB	2	sc-150	ChIP AMpure XP	GSM1010850/GSM1010878
CHD1	SYDH/Broad	2	A301-218A	IgG-mus/Std	GSM935301/GSM1003575
CHD2	SYDH	2	ab68301	IgG-mus/IgG-rab	GSM935378/GSM935502
CREB1	HAIB	2	sc-240	ChIP AMpure XP	GSM1010760/GSM1010890
CTCF	Broad	2	07-729	Std	GSM733752/GSM733719/ (HeLa GSM733785)
CUX1 (Cdpsc)	SYDH	2	sc-6327	IgG-mus/IgG-rab	GSM1003604/GSM1003622
E2F4	SYDH	2	sc-866	IgG-mus/UCDavis Input Control	GSM935330/GSM935330
EGR1	HAIB	2	sc-110	biorupter	GSM803434/GSM803414
ELK1	SYDH	2	1277-1	IgG-mus/IgG-rab	GSM935345/GSM1003620
EP300	SYDH	2	sc-585	IgG-mus/IgG-rab	GSM935559/GSM935401
ETS1	HAIB	2	sc-350	PCR 1-round/biorupter	GSM803510/GSM803442
EZH2	Broad	2	39875	Std	GSM1003498/GSM1003576
FOS (Cfos)	SYDH	3	sc-7202	Std	GSM935409/GSM935355
GABPA	HAIB	2	sc-28312	PCR 2-round/biorupter	GSM803356/GSM803524
JUND	SYDH	2	sc-74	IgG-rab	GSM1003634/GSM935569
MAFK	SYDH	2	ab50322	IgG-mus/IgG-rab	GSM1003616/GSM935311
MAX	SYDH	2	sc-197	IgG-mus/IgG-rab	GSM935518/GSM935344
MAZ	SYDH	2	ab85725	IgG-mus/IgG-rab	GSM935283/GSM935337
MEF2A	HAIB	2	sc-313	PCR 1-round/biorupter	GSM803511/GSM803379
MXI1	SYDH	2	AF4185	IgG-mus/IgG-rab	GSM935431/GSM935497
MYC (Cmyc)	UTA	2	sc-764	UTA Input Control	GSM822290/GSM822310
NFYA	SYDH	2	NF-YA	IgG-mus/Std/ (HeLa IgG-rab)	GSM935506/GSM935433/ (HeLa GSM935508)

NFYB	SYDH	2	NF-YB	IgG-mus/Std/ (HeLa IgG-rab)	GSM935507/GSM935429/ (HeLa GSM935408)
NR2C2 (Tr4)	SYDH	2	TR4	Std/UCDavis Input Control	GSM935480/GSM935374
NRF1	SYDH	2	ab55744	IgG-mus/IgG-rab	GSM935309/GSM935361
PML	HAIB	2	sc-71910	ChIP AMpure XP	GSM1010771/GSM1010722
PolII	UTA	2	MMS-126R	UTA Input Control	GSM822270/GSM822275
RAD21	SYDH	2	ab992	IgG-rab/Std	GSM935332/GSM935319
RCOR1 (Corest)	SYDH	2	sc30189	IgG-mus/IgG-rab	GSM935583/GSM935385
RFX5	SYDH	2	200-401-194	IgG-mus/IgG-rab	GSM935556/GSM935565
SIX5	HAIB	2	sc-55706	PCR 1-round	GSM803338/GSM803383
SMC3	SYDH	2	ab9263	IgG-mus/IgG-rab	GSM935376/GSM935310
SP1	HAIB	2	sc-17824	PCR 1-round	GSM803363/GSM803505
SPI1 (Pu1)	HAIB	3/2	sc-22805	PCR 1-round	GSM803531/GSM803384
SRF	HAIB	2	sc-335	biorupter	GSM803477/GSM803520
STAT5A	HAIB	2	sc-74442	ChIP AMpure XP	GSM1010721/GSM1010877
TAF1	HAIB	2	sc-735	PCR 1-round / biorupter	GSM803537/GSM803431
TBL1XR1	SYDH	2	ab24550	IgG-mus/IgG-rab	GSM935653/GSM935575
TBP	SYDH	2	ab62126	IgG-mus	GSM935277/GSM935495
USF1	HAIB	2	sc-229	PCR 2-round / biorupter	GSM803347/GSM803441
USF2	SYDH	2	ab60931	IgG-mus/IgG-rab/ (HeLa IgG-mus)	GSM935558/GSM935356/ (HeLa GSM935561)
ZBTB33	HAIB	2	sc-23871	PCR 1-round	GSM803392/GSM803504
ZNF143	SYDH	2	16618-1-AP	Std/IgG-rab	GSM935613/GSM935568
ZNF274	SYDH	2	H00010782-A01	Std/UCDavis Input Control	GSM935349/GSM935479
ZNF384	SYDH	2	HPA004051	IgG-mus/IgG-rab	GSM1003602/GSM1003621

*Lab abbreviation: HAIB - HudsonAlpha, SYDH - Stanford/Yale/USC/Harvard, Broad - Broad Institute, UTA - UT Austin

Table S2: Details of downloaded data control (GM12878/K562)

Lab	Control/Lab Protocol	Detail	GEO Sample Accession
HAIB	PCR 1-round	one 15-cycle round of PCR (Myers)	GSM803351/GSM803352
	PCR 2-round	a 25-cycle round of PCR and an additional 15-cycle round of PCR after gel size selection (Myers)	GSM803346/ -
	biorupter	2×10^7 cells, fragmentation by bioruptor, one 15-cycle round of PCR (Myers)	GSM803413/GSM803468
	ChIP AMpure XP	Faster ChIP protocol and AMpure XP size selection for ChIP-Seq (Myers)	GSM1010867/GSM1010894
SYDH	Std	Standard input signal for most experiments	GSM935413/GSM935363
	IgG-mus	Input signal from Normal Mouse IgG ChIP-Seq	GSM935390/GSM935631/ (HeLa GSM935535)
	IgG-rab	Input signal from Normal Rabbit IgG ChIP-Seq	GSM935450/GSM935618/ (HeLa GSM935339)
	UCDavis Input Contro	Input library was prepared at UC Davis	- /GSM935601
Broad	Std	Standard input signal for most experiments	GSM733742/GSM733780
UTA	UTA Input Control	Standard input signal for most experiments	GSM822292/GSM822293/ (HeLa GSM733659)

Table S3: Top five most significant GO categories for each of the active cluster in both cell lines.

	GM12878			K562		
	GO Term	*FE	**BH	GO Term	*FE	**BH
Cluster I	translational elongation	12.50	1.25E-23	translational elongation	9.47	3.11E-13
	RNA splicing	4.87	7.31E-11	RNA splicing	4.12	8.41E-06
	regulation of protein ubiquitination	8.57	4.1E-08	RNA processing	2.42	1.34E-04
	RNA splicing, via transesterification reactions	6.83	4.3E-08	macromolecular complex subunit organization	2.57	1.63E-04
	negative regulation of molecular function	4.62	4.44E-08	mRNA processing	3.32	1.66E-04
Cluster NFY	transcription	2.82	3.60E-15	transcription	3.00	4.88E-17
	regulation of transcription	2.29	6.68E-12	regulation of transcription	2.38	1.06E-12
	chromosome organization	4.16	1.49E-11	cell cycle process	3.49	6.39E-10
	chromatin organization	4.17	2.22E-08	cell cycle	2.92	9.82E-10
	chromatin assembly or disassembly	7.39	4.49E-08	M phase	3.90	2.11E-08
Cluster USF	transcription	2.36	3.83E-08	transmembrane transport	4.34	3.64E-03
	negative regulation of gene expression	4.21	4.06E-08			
	regulation of transcription	2.06	6.58E-08			
	negative regulation of transcription	4.03	2.38E-06			
	negative regulation of macromolecule metabolic process	3.18	2.63E-06			
Cluster CTCF	response to wounding	5.02	2.44E-04	negative regulation of cell death	8.40	1.43E-05
	inflammatory response	6.05	4.72E-03	anti-apoptosis	12.93	1.89E-05
	protein complex assembly	3.00	7.08E-03	negative regulation of programmed cell death	8.4	2.01E-05
	protein complex biogenesis	3.00	7.08E-03	regulation of cell death	4.98	3.38E-05
	macromolecular complex assembly	2.68	9.09E-03	negative regulation of apoptosis	8.50	3.77E-05

*FE: Fold Enrichment **BH: Benjamin Hochberg's correction

Supplementary Figures

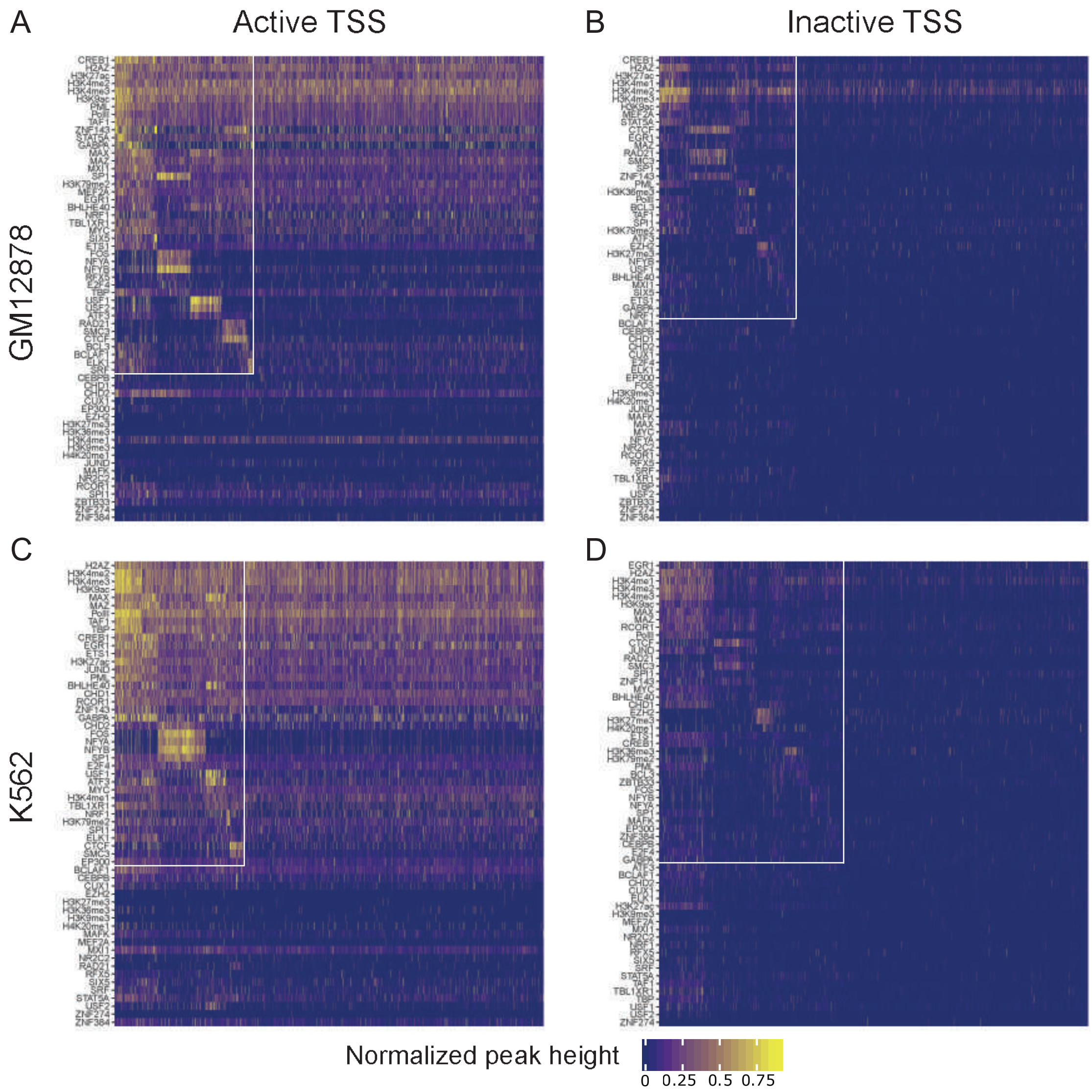

Figure S1: **TSSs that are assigned to clusters according to biclustering algorithm.** Heatmaps for biclustering result of active and inactive TSS of both cell-lines. ChIP-seq tracks (rows) and TSSs(columns) are ordered according to the biclustering result. TSSs and ChIP-seq experiments that are clustered by the biclustering algorithm are highlighted by the boxes delineated at the top left. In GM12878, 41 rows and 1957 columns are selected in active TSSs (A), 34 rows and 2189 columns are selected in inactive TSSs (B), In K562, 38 rows and 1257 columns are selected in active TSSs (C), 39 rows and 4129 columns are selected in inactive TSSs (D).

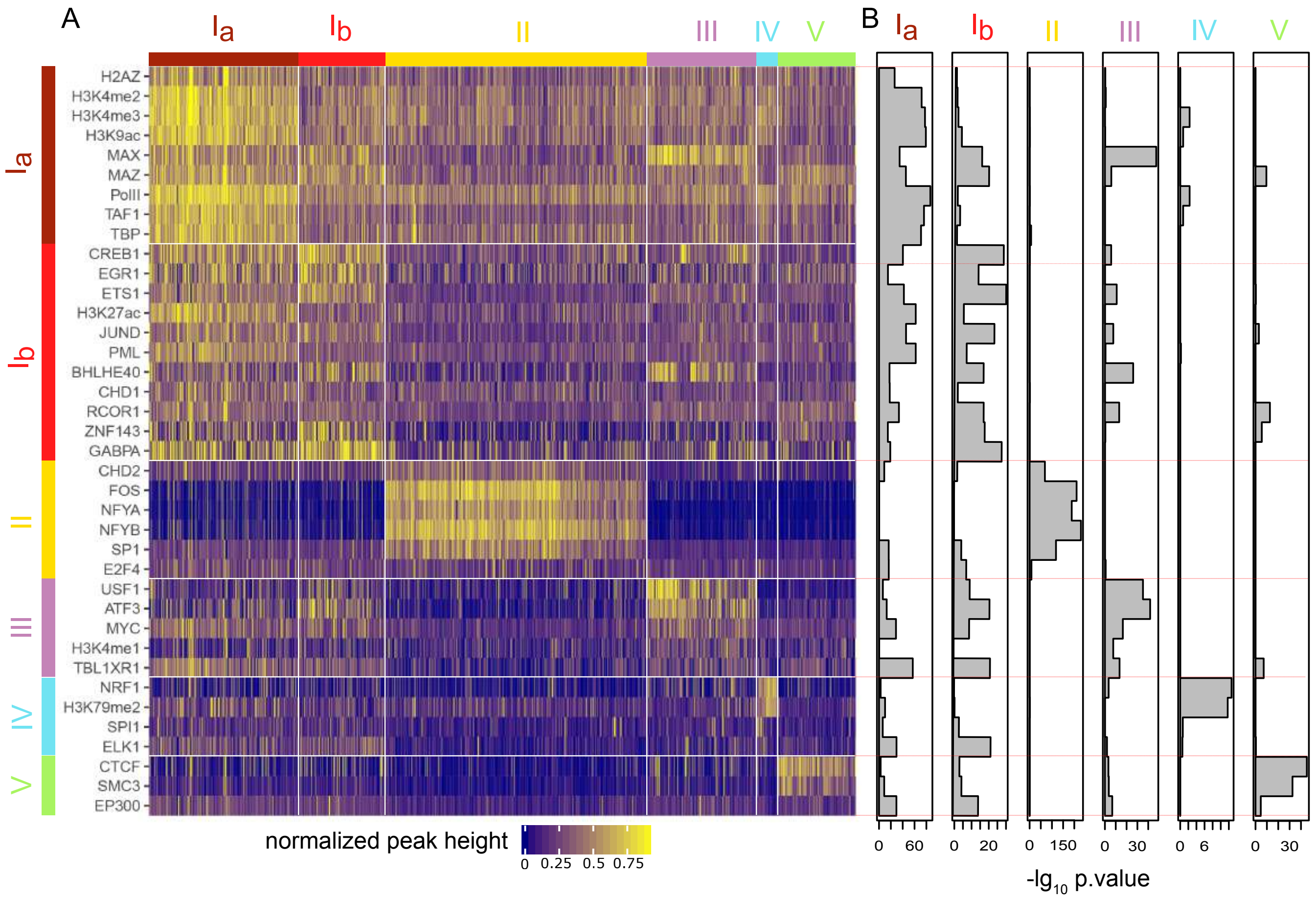

Figure S2: **Visualization of biclustering result of active TSS in K562 cell-line.** A: ChIP-seq tracks (rows) and promoters (columns) are ordered according to the biclustering and displayed as a heatmap. The heatmap color corresponds to normalized peak height (see Methods) B: Result of t-test measuring cluster association for each row. The bars extend to the right to a height of the negative logarithm base 10 of the p-value. A high t-test value for a row and a certain cluster indicates that this row's HM/TF is enriched in the respective cluster.

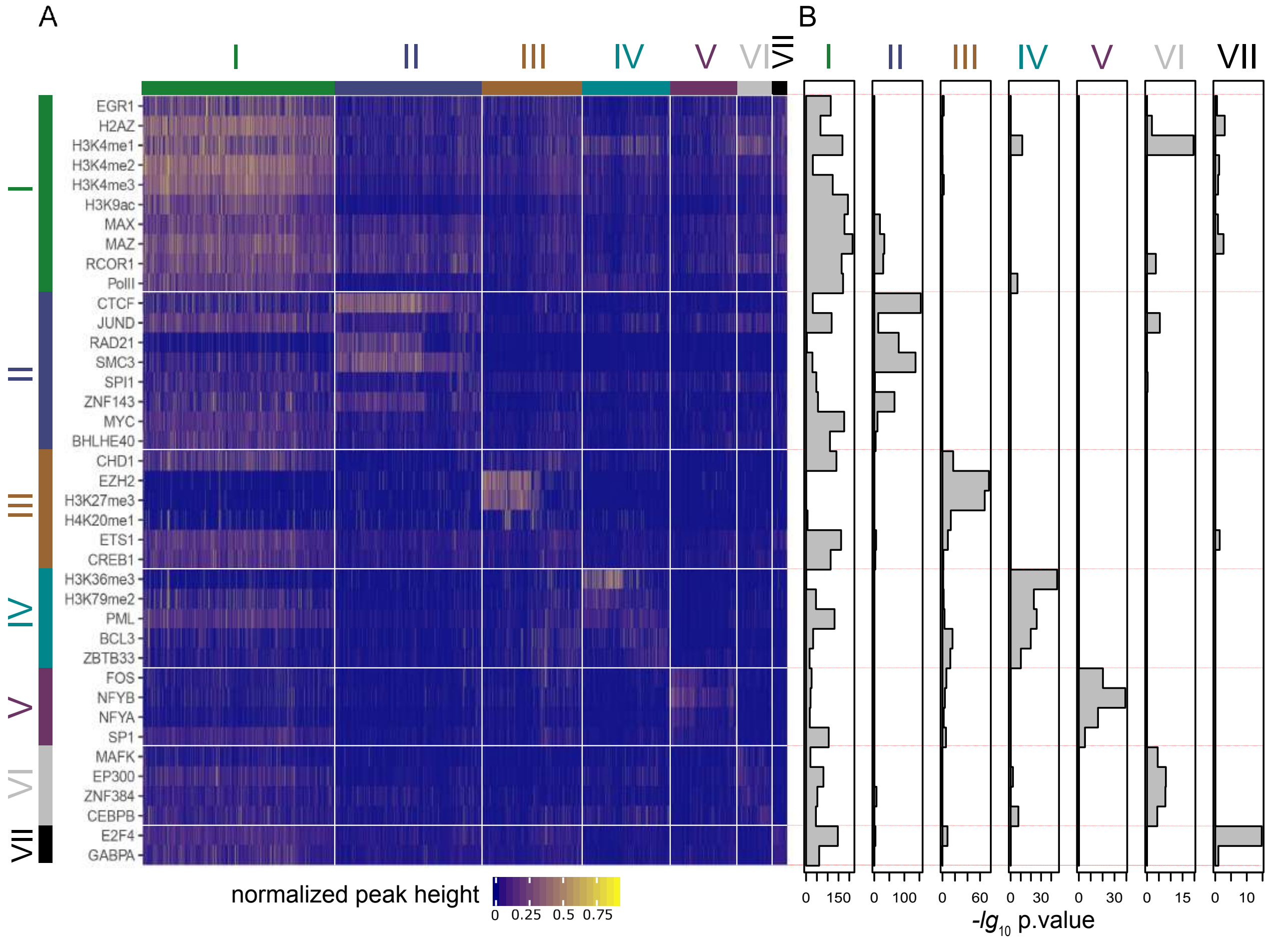

Figure S3: **Visualization of biclustering result of inactive TSS in K562 cell-line.** A: ChIP-seq tracks (rows) and promoters (columns) are ordered according to the biclustering and displayed as a heatmap. The heatmap color corresponds to normalized peak height (see Methods) B: Result of t-test measuring cluster association for each row. The bars extend to the right to a height of the negative logarithm base 10 of the p-value. A high t-test value for a row and a certain cluster indicates that this row's HM/TF is enriched in the respective cluster.

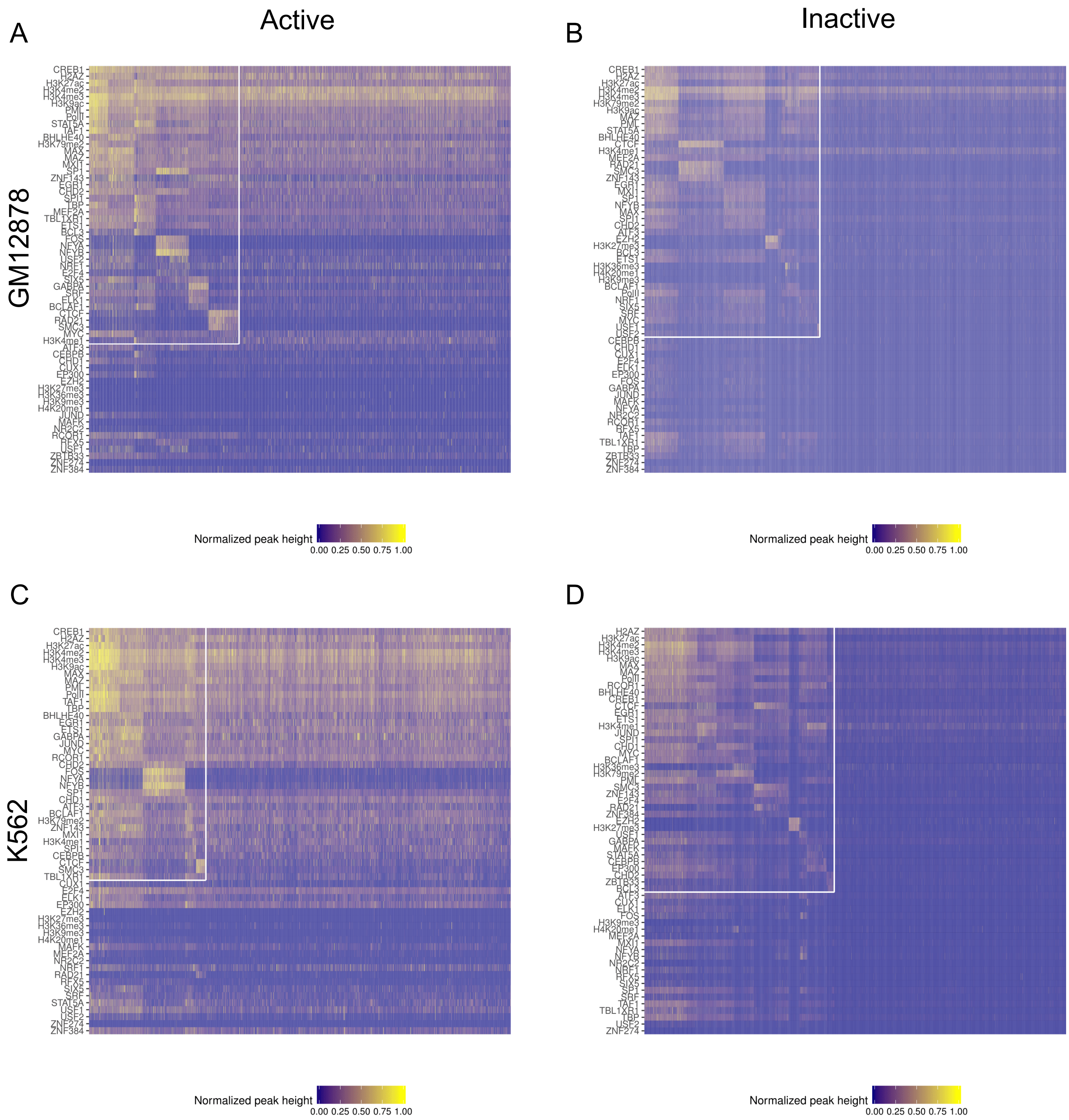

Figure S4: **TSSs that are assigned to clusters according to biclustering algorithm based on CAGE tag annotation** Heatmaps for biclustering results of TSS. ChIP-seq tracks (rows) and TSSs(columns) are ordered according to the biclustering result. TSSs and ChIP-seq experiments that are clustered by the biclustering algorithm are on the top-left of the matrix. A) Active TSSs in GM12878 cell-line, 41 rows and 6867 columns are selected. B) Inactive TSSs in GM12878 cell-line. 40 rows and 12740 columns are selected. C) Active TSSs in K562 cell-line. 36 rows and 3559 columns are selected. D) Inactive TSSs in K562 cell-line. 39 rows and 18469 columns are selected.

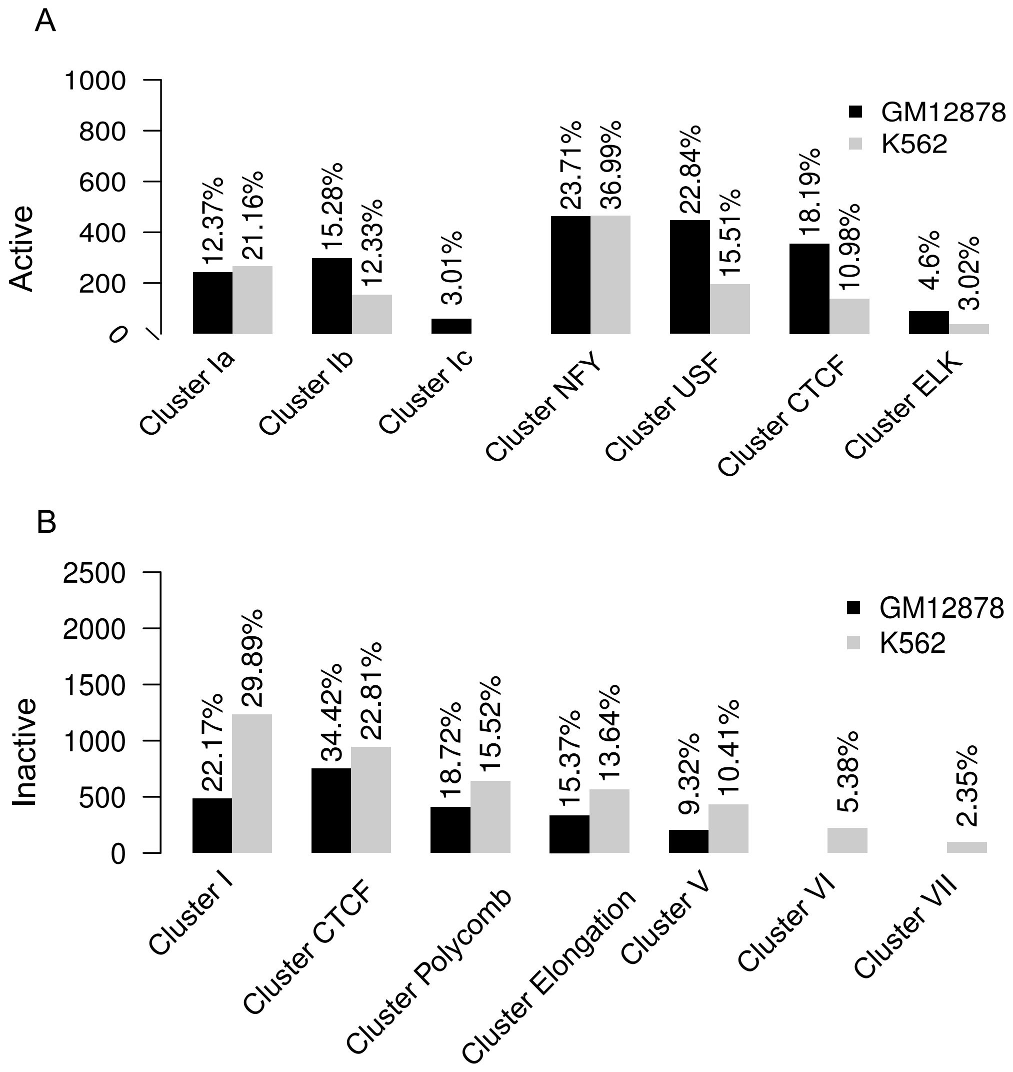

Figure S5: **Bar plot for the proportion of each cluster among all assigned promoters.** A and: Bar plot for the number of each cluster in all assigned active/inactive promoters in GM12878 cell-line and active/inactive promoters in K562 cell-line, respectively. Proportion of each of the cluster are showed on the top of each bar.

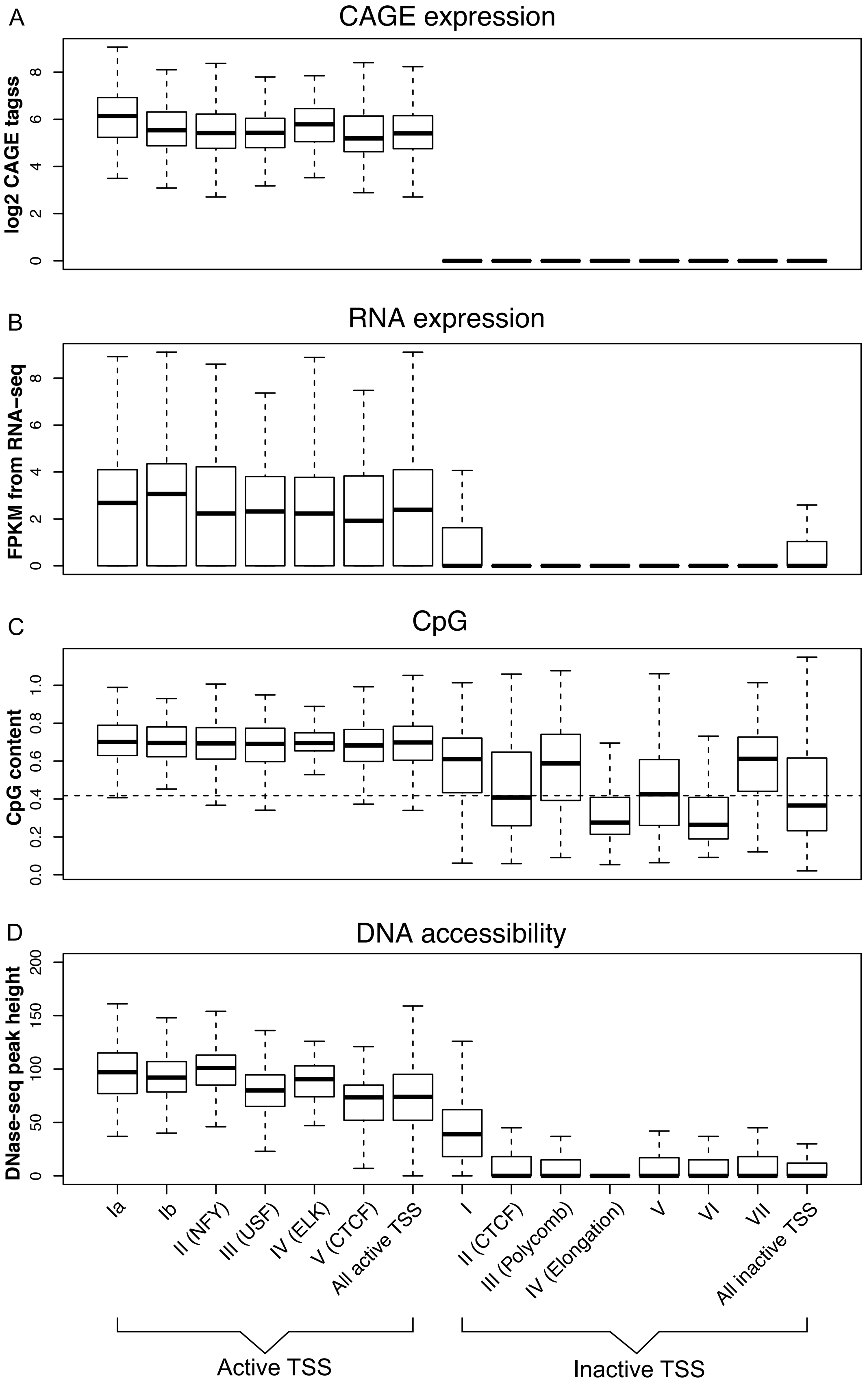

Figure S6: **Expression measures and possible covariates associated to the individual clusters in K562 cell line.** Box plots of values of (A) logarithm of number of CAGE tags. This is the criterion that served to determine active and silent promoters. (B) FPKM of RNA-seq for the target genes of the promoters in the cluster. (C) CpG contents in promoter region. The dashed line is the cutoff for defining high/low CpG-contents human promoter. (D) Height of DNase accessibility peak in the promoters for each cluster. Cluster identifiers given at the bottom of the figure refer to all parts of the figure.

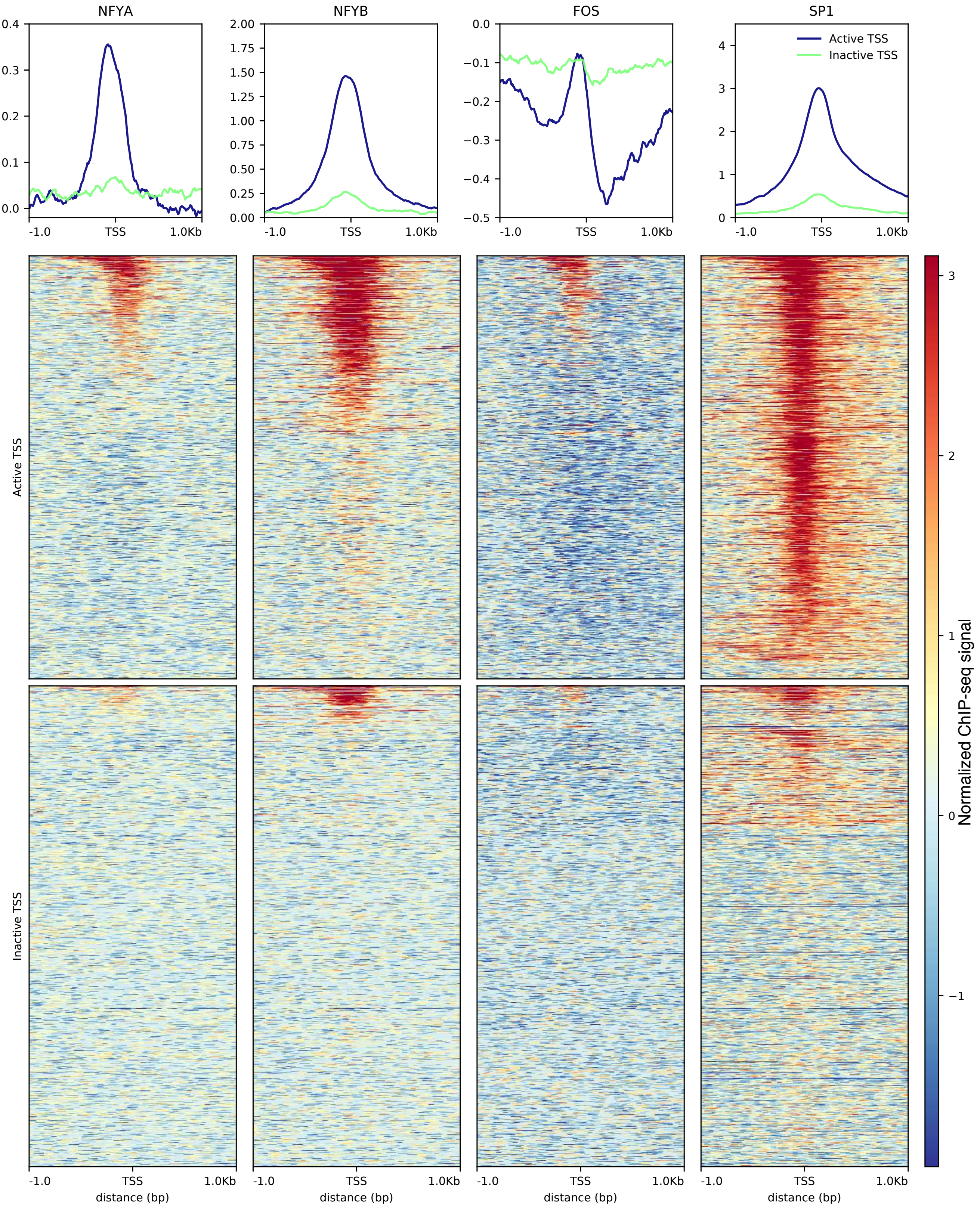

Figure S7: **NFY co-binding pattern in GM12878 cell-line.** Density plots and coverage patterns for ChIP-seq signals of NFYA/B, FOS, and SP1 in +/- 1Kbp window of TSSs in active (upper boxes) and inactive TSSs (lower boxes) in GM12878 cell-line. TSSs are ordered by the peak height of NFYA and then NFYB, FOS, SP1. TSSs without peaks of any of these four ChIP-seq are not shown.

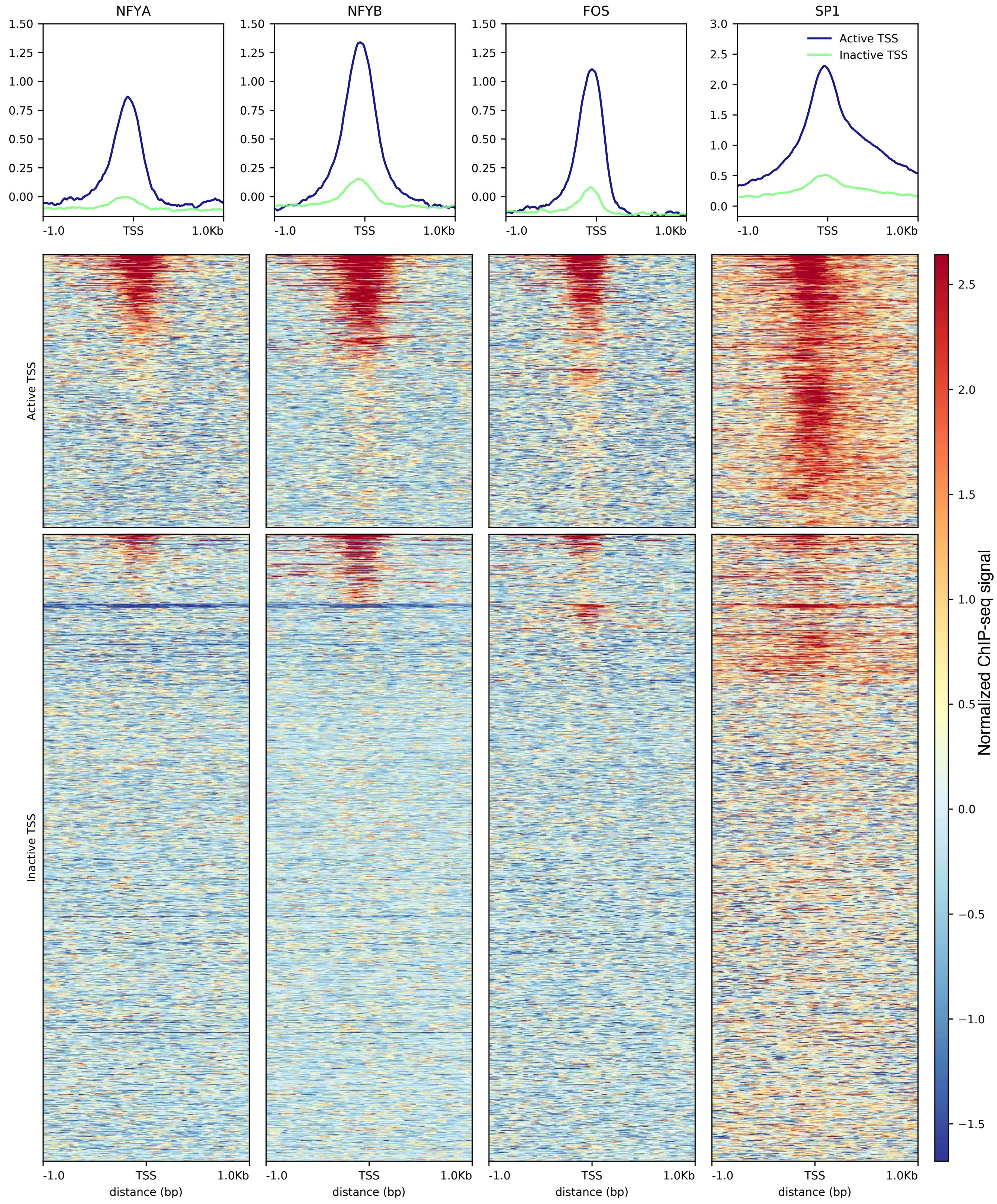

Figure S8: **NFY co-binding pattern in K562 cell-line.** Density plots and coverage patterns for ChIP-seq signals of NFYA/B, FOS, and SP1 in +/- 1Kbp window of TSSs in active (upper boxes) and inactive TSSs (lower boxes) in K562 cell-line. TSSs are ordered by the peak height of NFYA and then NFYB, FOS, SP1. TSSs without peaks of any of these four ChIP-seq are not shown.

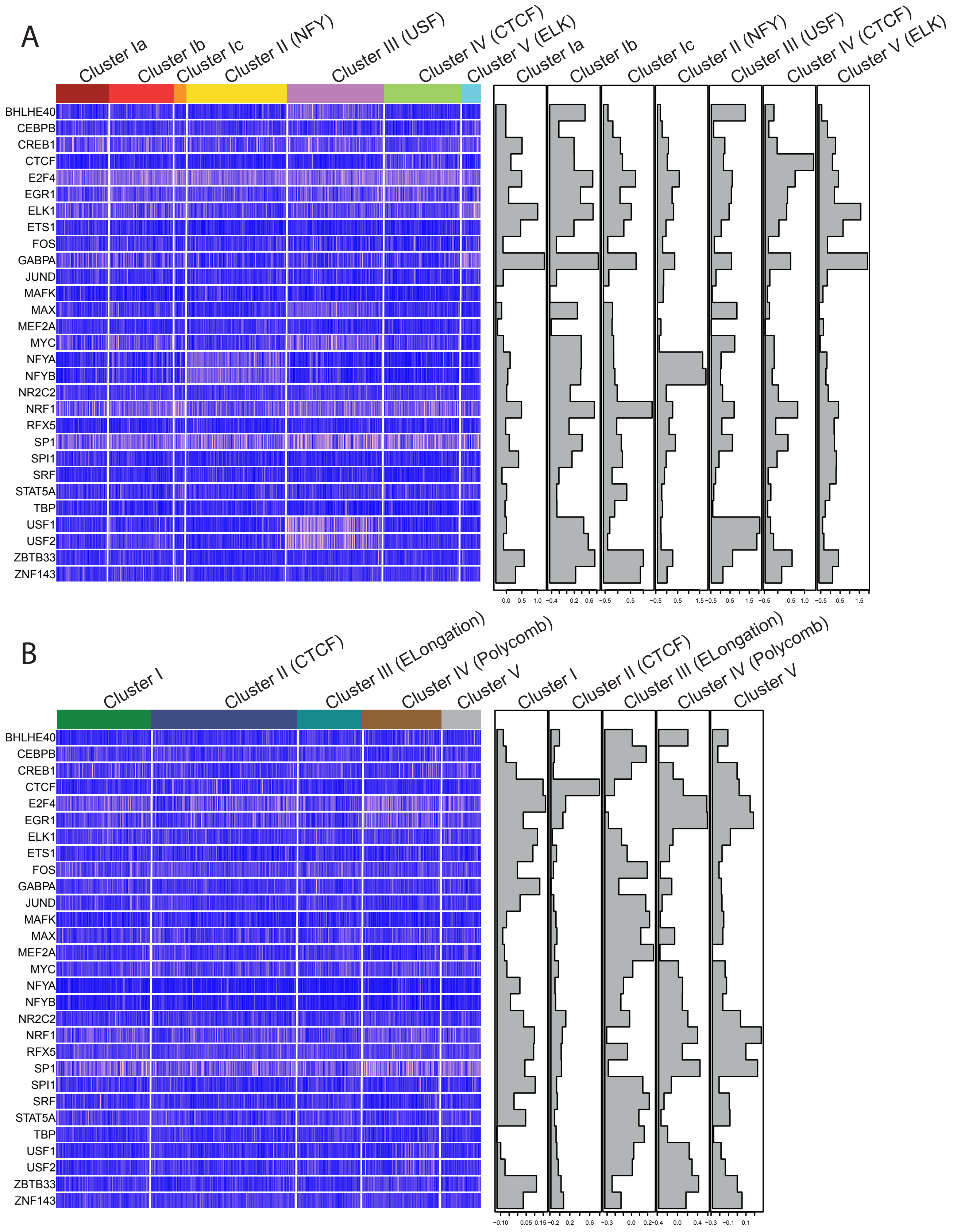

Figure S9: **TF motif hits in promoters in GM12878 cell-line.** Promoters were annotated with TF motif hits for all TFs whose ChIP-seq is included in our analysis and where a motif is available in Jaspar 2014. Heatmap color corresponds to motif hit score from MAST (see Methods in the main text). TSSs are ordered in columns as Figure 1 and 2 in the main text and with the cluster assignment from the GM12878 cell-line annotated on the top. The graphs on the right indicate the average motif hit score per cluster. Part A is for active promoters, B is for inactive promoters.

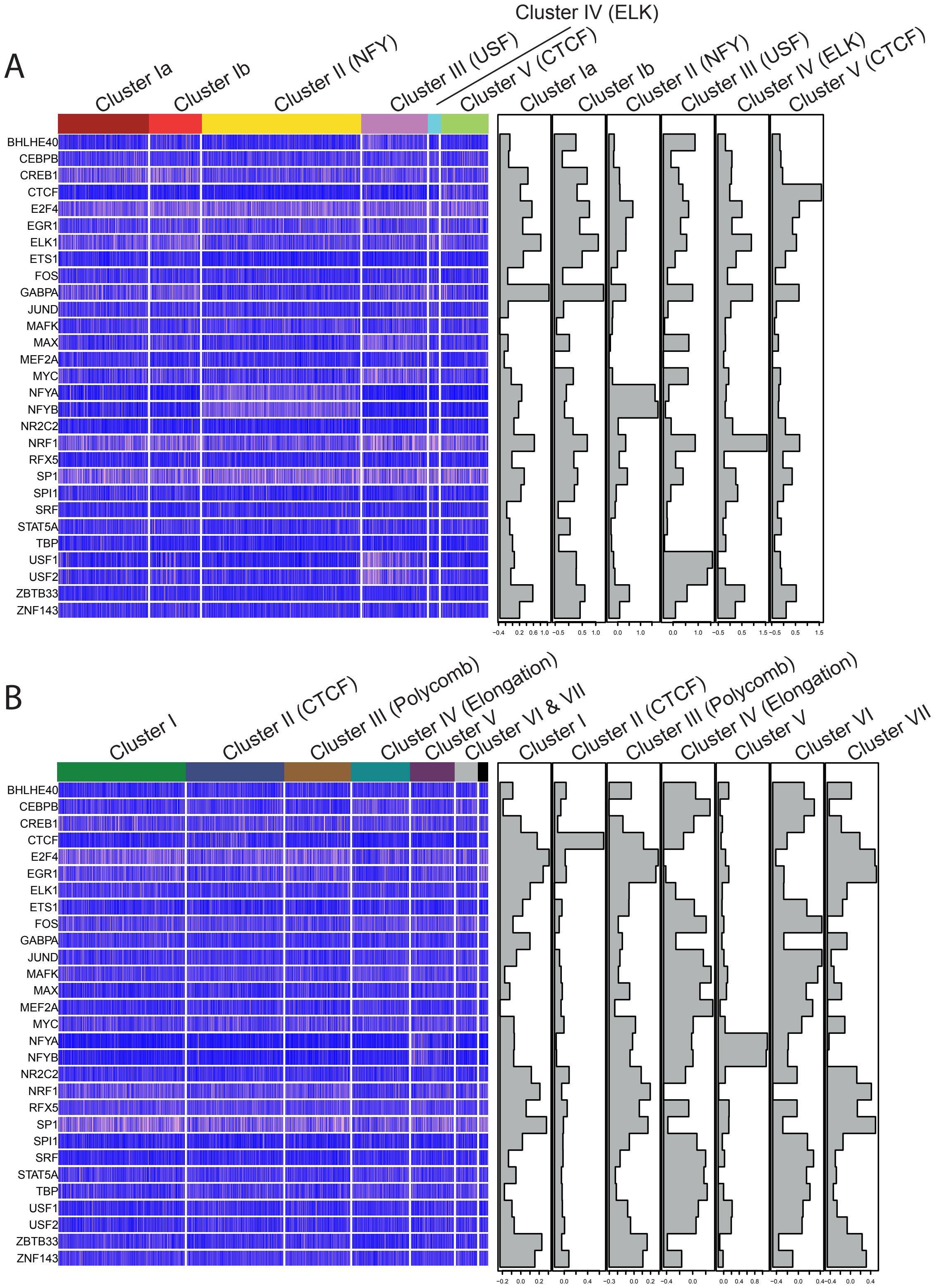

Figure S10: **TF motif hits in promoters in K562 cell-line.** Promoters were annotated with TF motif hits for all TFs whose ChIP-seq is included in our analysis and where a motif is available in Jaspar 2014. Heatmap color corresponds to motif hit score from MAST (see Methods in the main text). TSSs are ordered in columns as Supplementary Figure 2 and 3 and with the cluster assignment from the K562 cell-line annotated on the top. The graphs on the right indicate the average motif hit score per cluster. Part A is for active promoters, B is for inactive promoters.

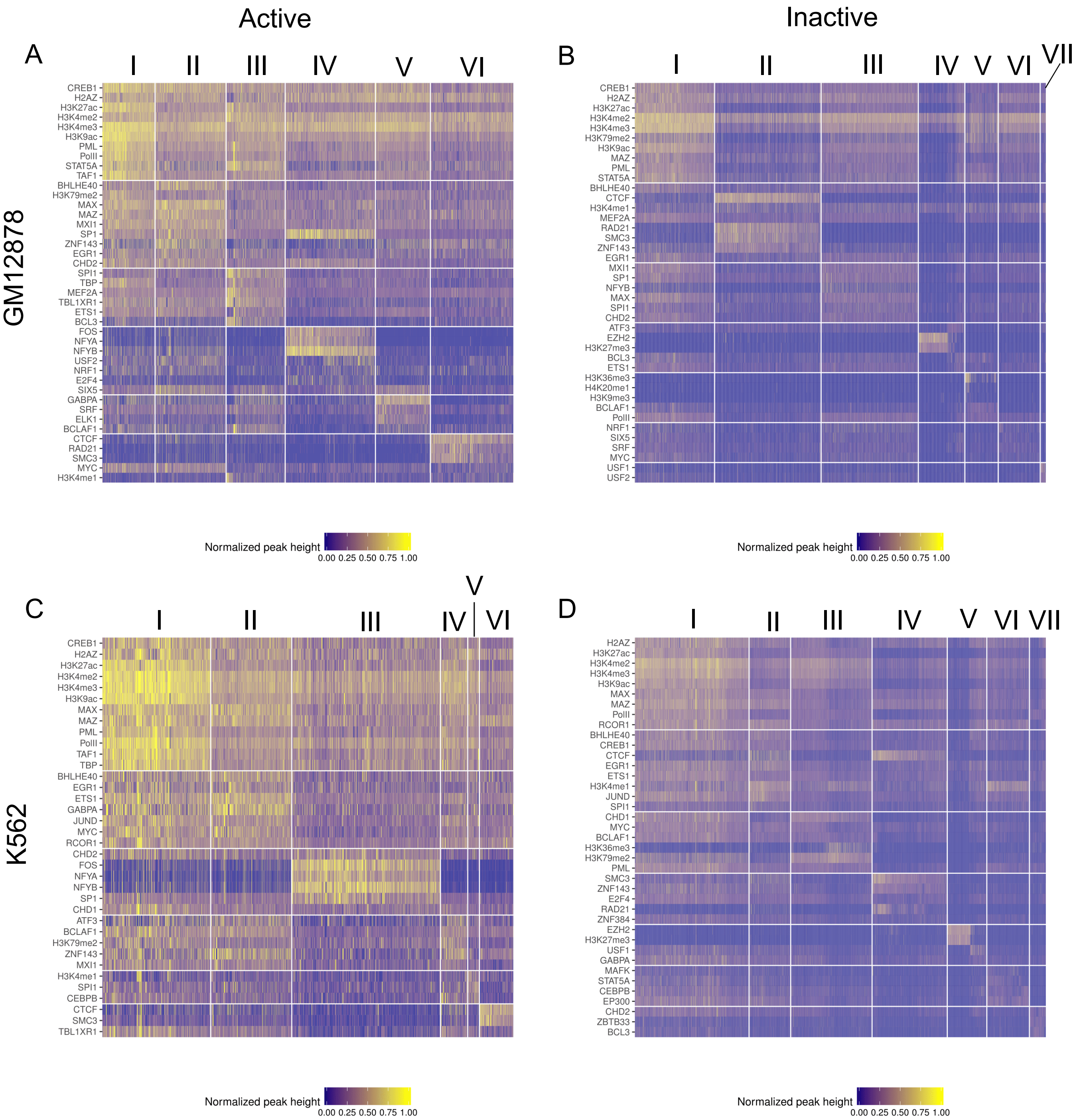

Figure S11: **Visualization of biclustering results based on CAGE tags.** ChIP-seq tracks (rows) and promoters (columns) are ordered according to the biclustering and displayed as a heatmap. The heatmap color corresponds to normalized peak height (see Methods). Clusters are numbered as Rome letters. A) Active TSS in GM12878 cell-line. B) Inactive TSS in GM12878 cell-line. C) Active TSS in K562 cell-line. D) Inactive TSS in K562 cell-line.

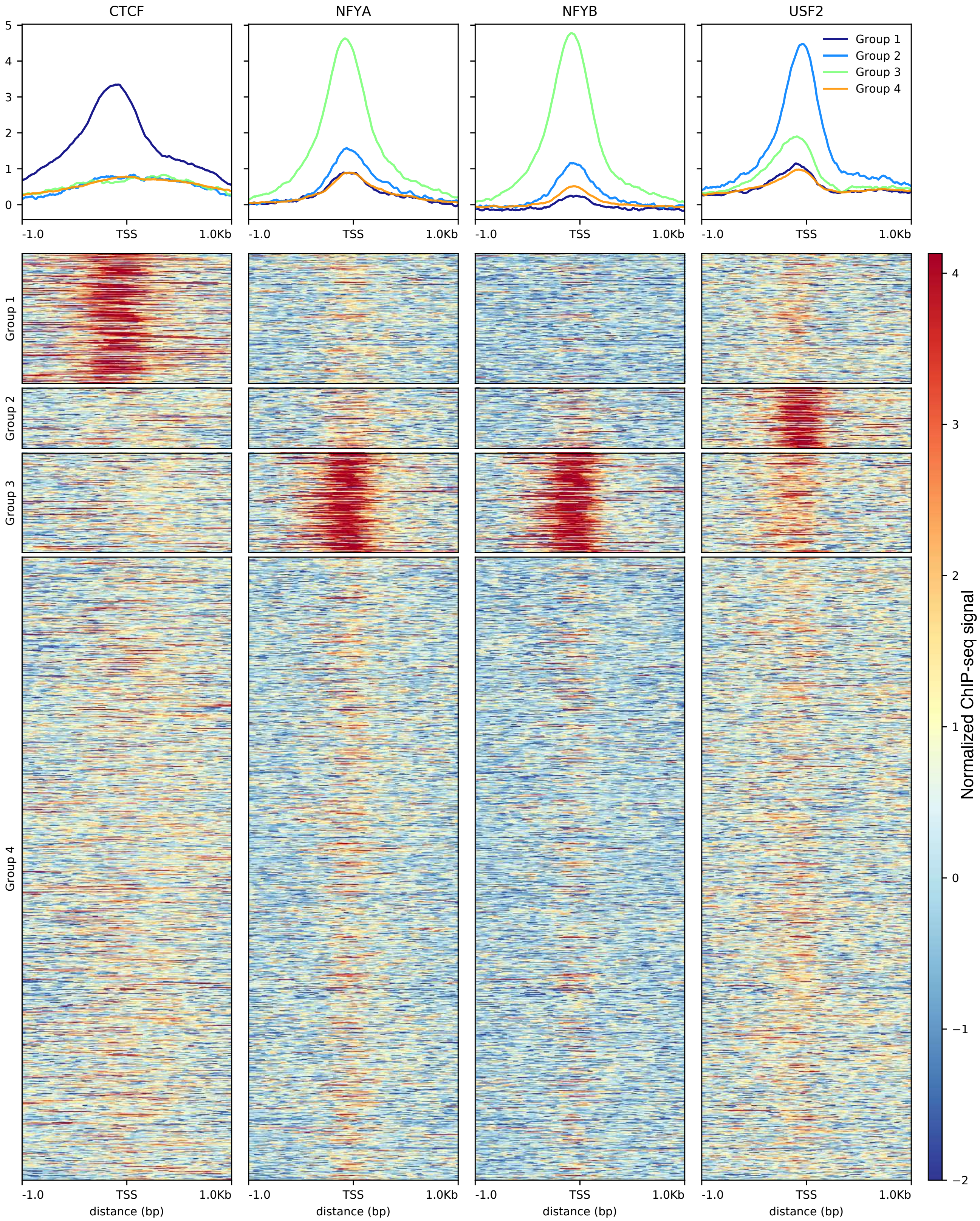

Figure S12: **Validation of NFY-, USF-, and CTCF-clusters in HeLa cells.** Coverage patterns of CTCF, NFYA/B and USF2 ChIP-seq signals in +/- 1Kbp window around TSSs in Hela cell-line. Promoters without called peaks (MACS) in all four ChIP-seqs were discarded. Promoters are clustered into four groups using the k-means clustering algorithm.

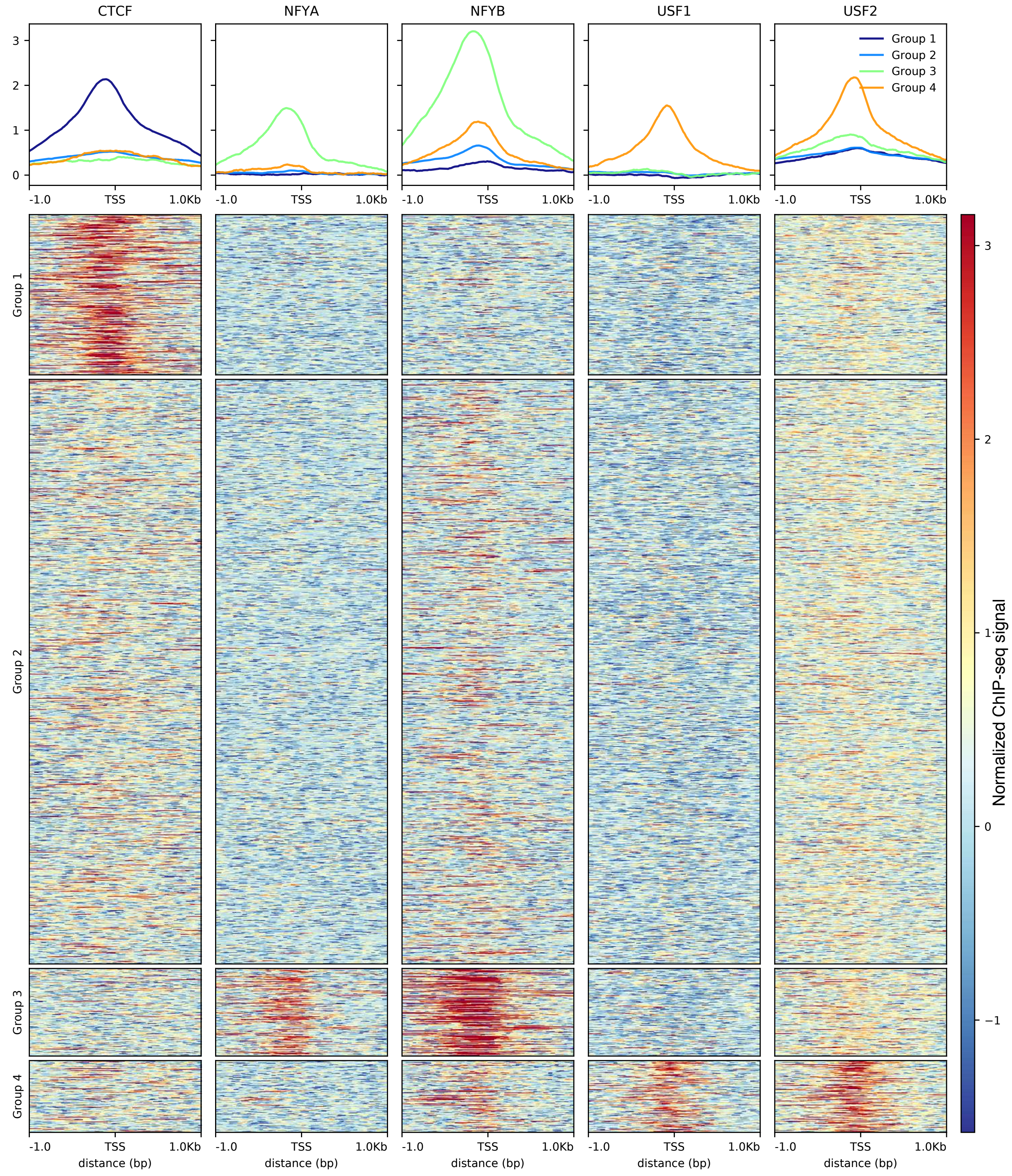

Figure S13: **Validation of NFY-, USF-, and CTCF-clusters in GM12878 cells based on CAGE TSS.** Coverage patterns of CTCF, NFYA/B and USF2 ChIP-seq signals in +/- 1Kbp window around TSSs in GM12878 cell-line. Promoters without called peaks (MACS) in all four ChIP-seqs were discarded. Promoters are clustered into four groups using the k-means clustering algorithm.

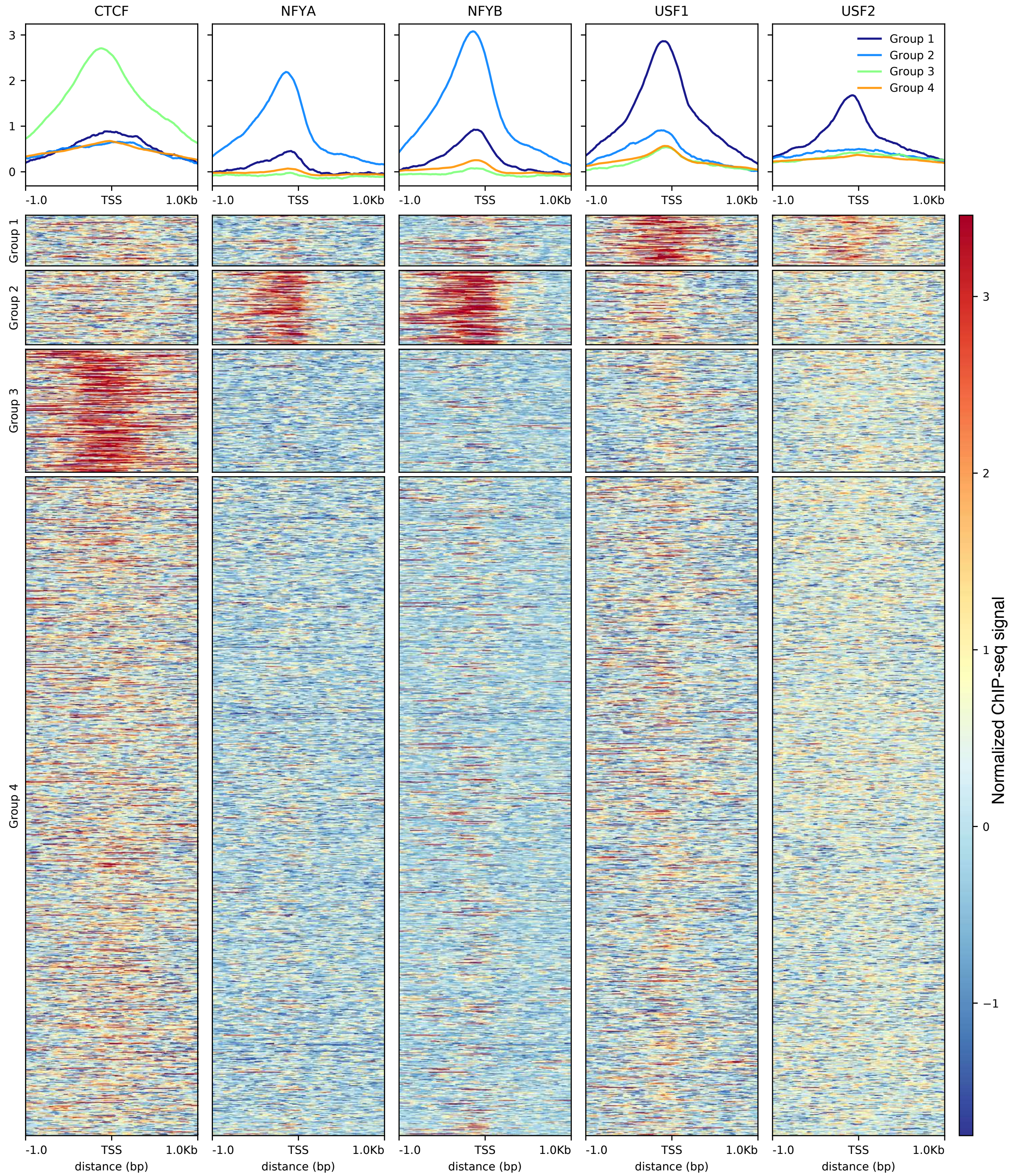

Figure S14: **Validation of NFY-, USF-, and CTCF-clusters in K562 cells based on CAGE TSS.** Coverage patterns of CTCF, NFYA/B and USF2 ChIP-seq signals in +/- 1Kbp window around TSSs in K562 cell-line. Promoters without called peaks (MACS) in all four ChIP-seqs were discarded. Promoters are clustered into four groups using the k-means clustering algorithm.

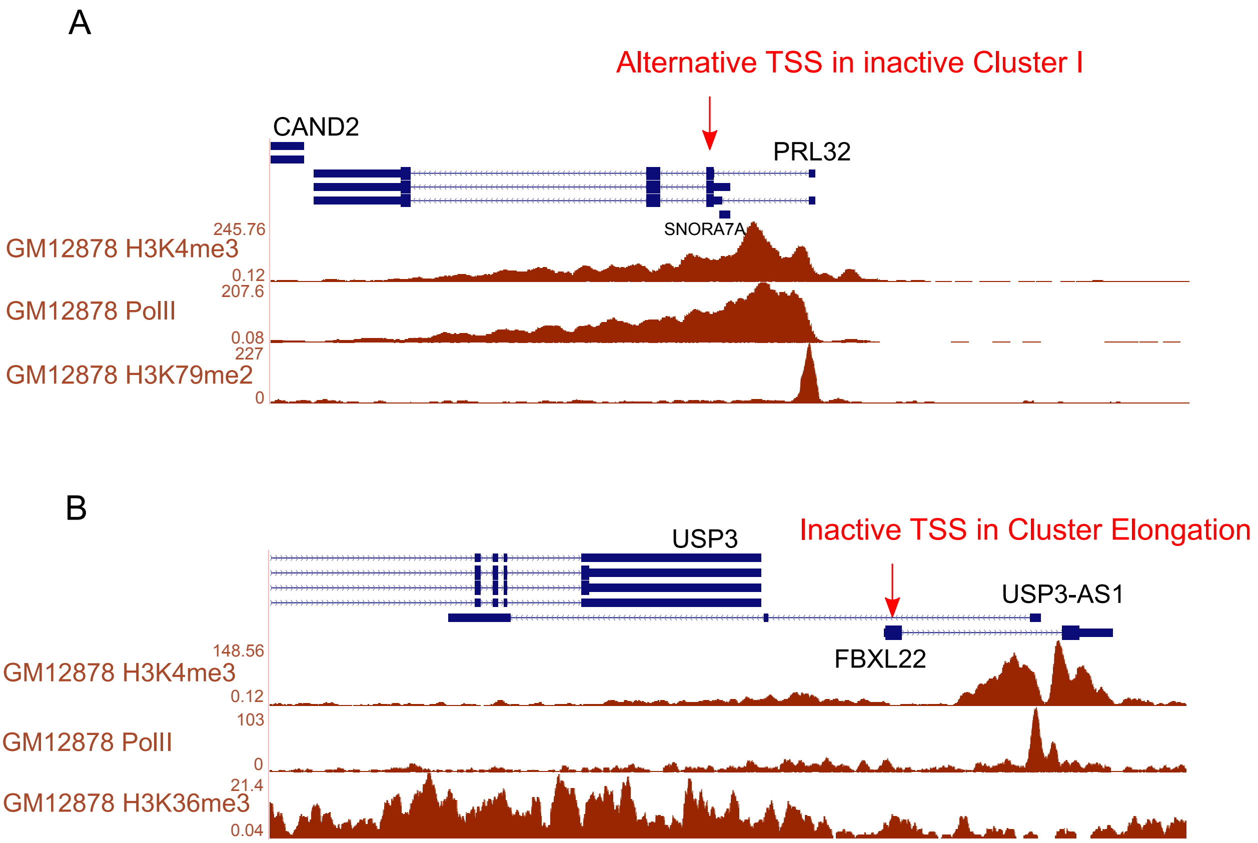

Figure S15: **Examples of inactive TSS embedded in an active gene.** A: Genome browser screen shot of TSS of gene Fbx22. H3K4me3, PolII and H3K36me3 tracks are shown in the figure. Fbx22 TSS is covered by H3K36me3 becasue it is in the gene body of USP3-AS1, which is transcribed. B: Genome browser screen shot of TSS of gene Rpl32. The downstream alternative promoter of Rpl32 is marked by H3K79me2 apparently because the upstream promoter of that gene is active.

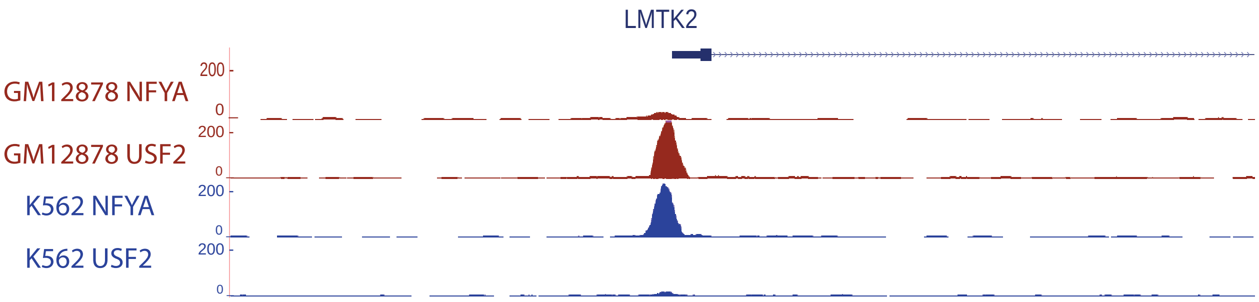

Figure S16: **Example of a promoter bound either by NFY or by USF in the two cell-lines.** Genome browser screen shot of the promoter of gene Lmtk2. NFYA and USF2 tracks in GM12878 (in orange) and K562 in(blue) are shown.

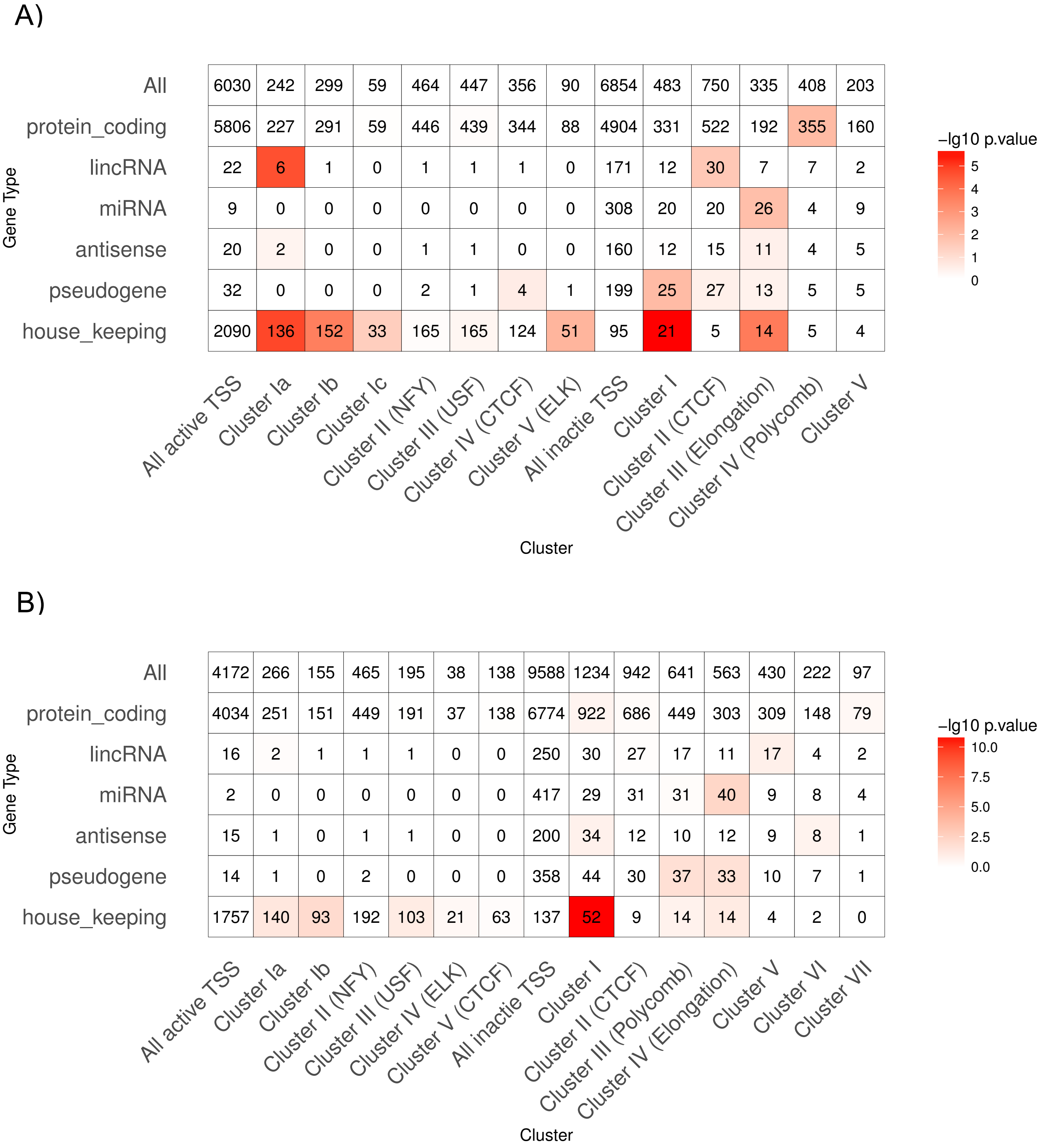

Figure S17: **Transcript type and function analysis for genes in each cluster.** Numbers of genes from each cluster with specific transcript types in GM12878 (A) and K562 (B). $-log10$ p-values of χ^2 test are shown as color gradient in the matrix.

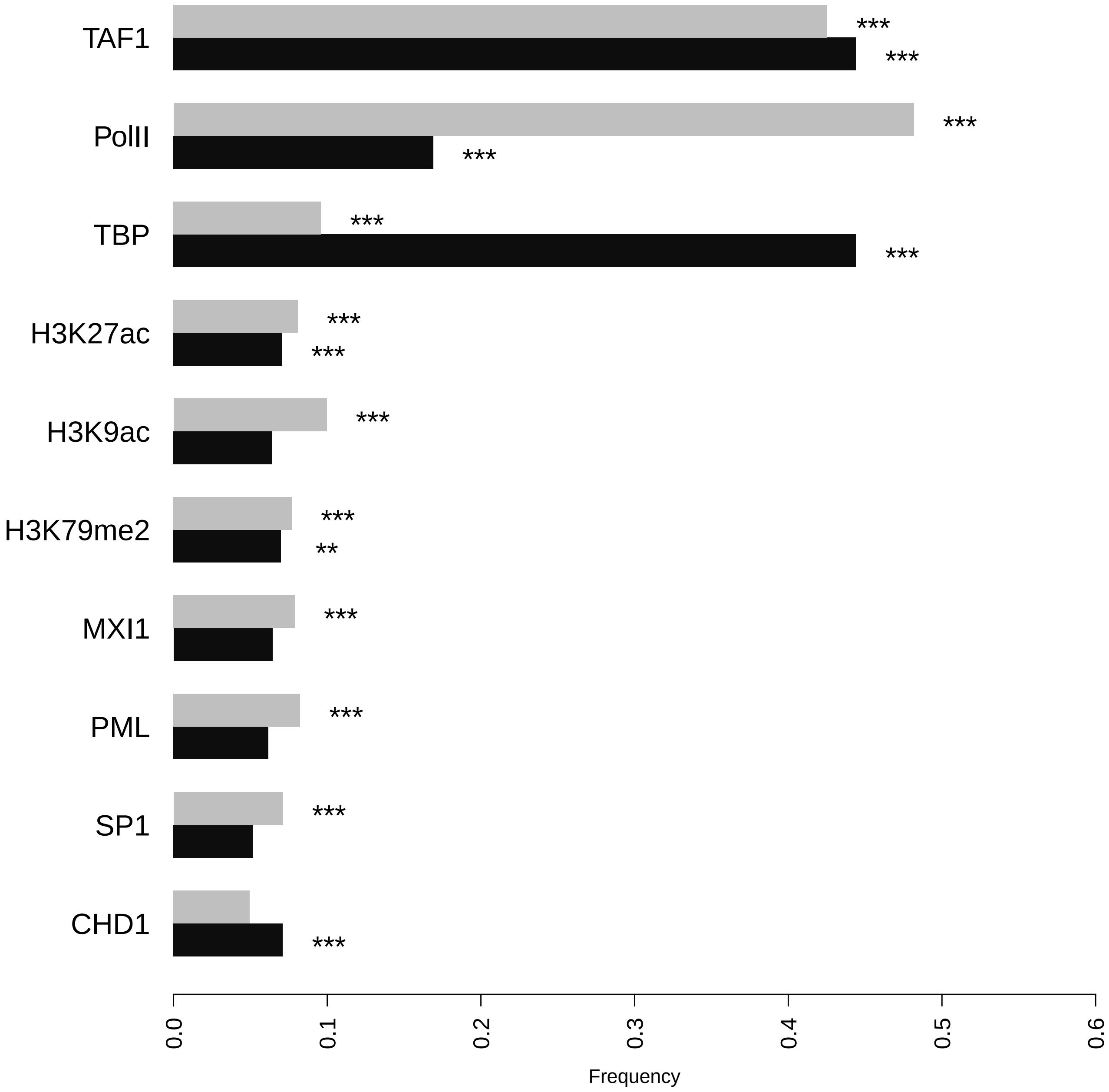

Figure S18: **Histone modifications and transcription factors that significantly contribute to the gene expression.** Bar plot of the frequency of appearance of ChIP-seq experiments in the "good sets" in GM12878 (gray) and K562 (black) cell line. In each of the cell line, ChIP-seq are ranked by the p-value of the hypergeometric test. In this figure, ChIP-seq are ordered by the sum of the rank in two cell lines.

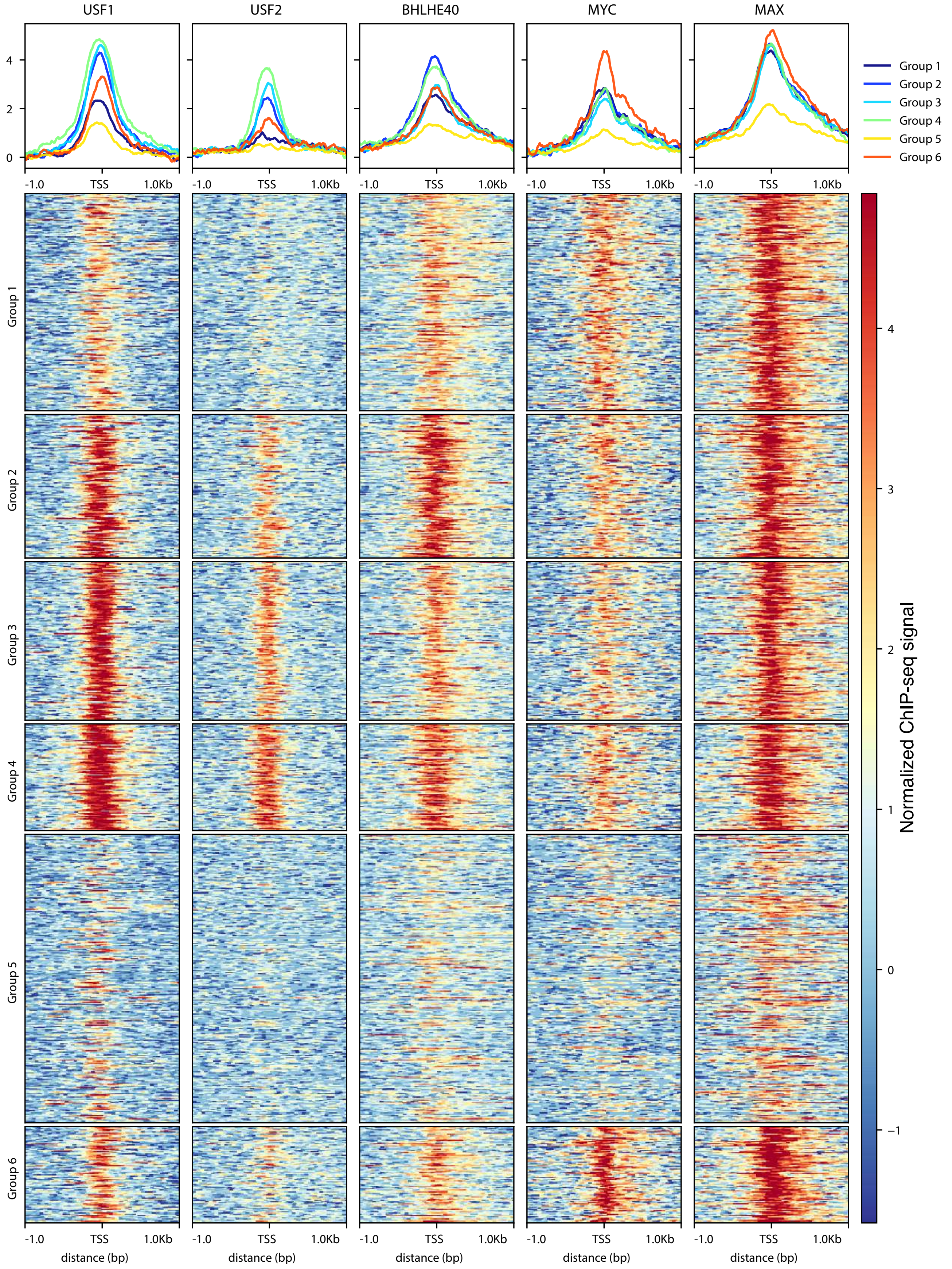

Figure S19: **Binding combinatorics in E-box containing promoters in K562 cell-line.** Density plots and coverage patterns for ChIP-seq signals of TFs recognizing the E-box (USF1/2, BHLHE40, MYC, MAX) in +/- 1Kbp window of the selected TSSs in GM12878 cell-line. Promoters are ordered in columns and grouped according to k-means clustering.

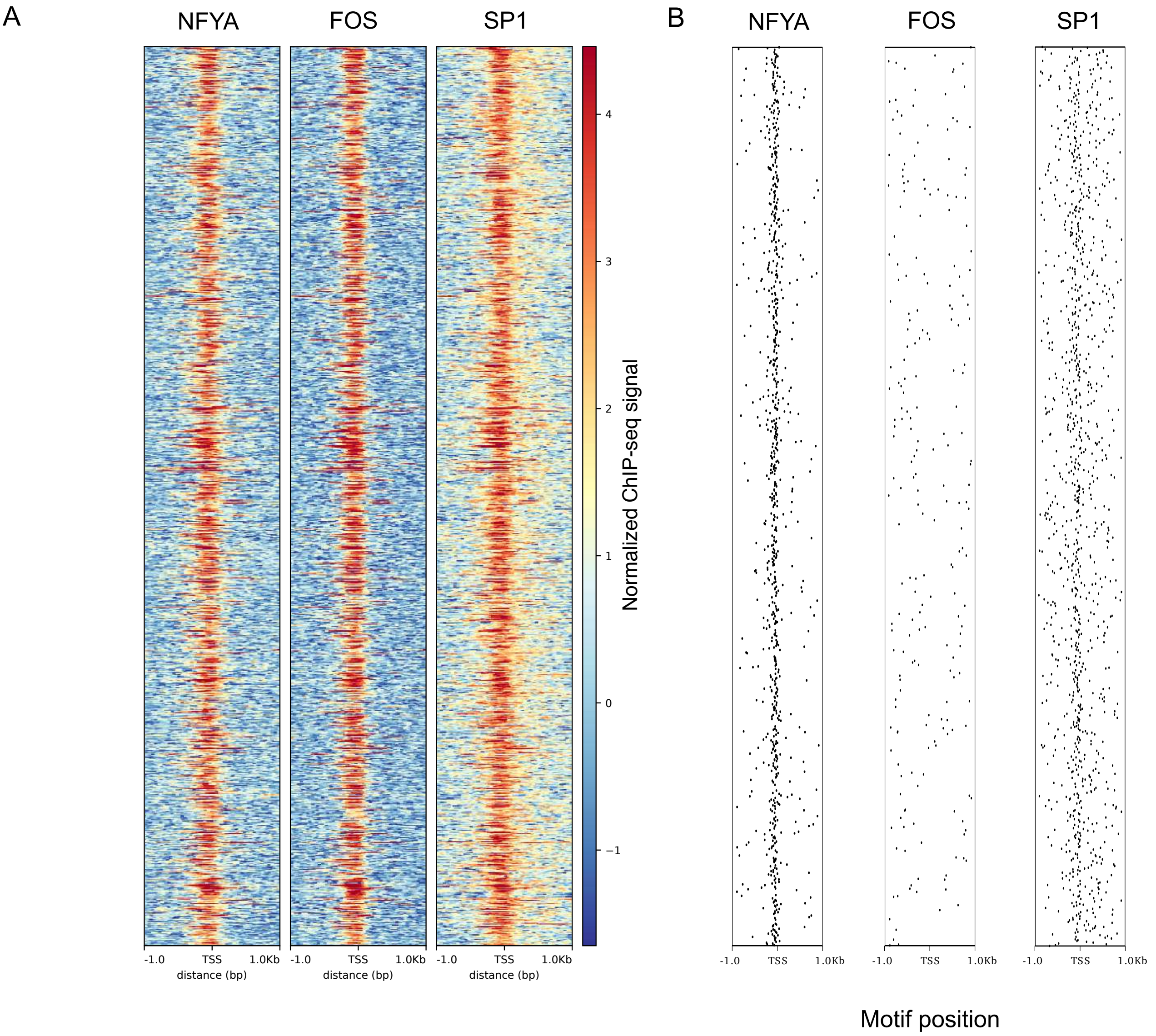

Figure S20: **Binding patterns of NFYA, FOS and SP1 compared to motif occurrence in K562 cell-line.** A: Coverage patterns for ChIP-seq signals of NFYA, FOS, and SP1 in a +/- 1Kbp window of selected TSSs in GM12878. B: Estimated motif position for the same TFs and TSS windows as A. While motif locations for NFYA and SP1 are concordant with the ChIP-seq signal, the FOS motif locations cannot explain the FOS ChIP-seq signal.

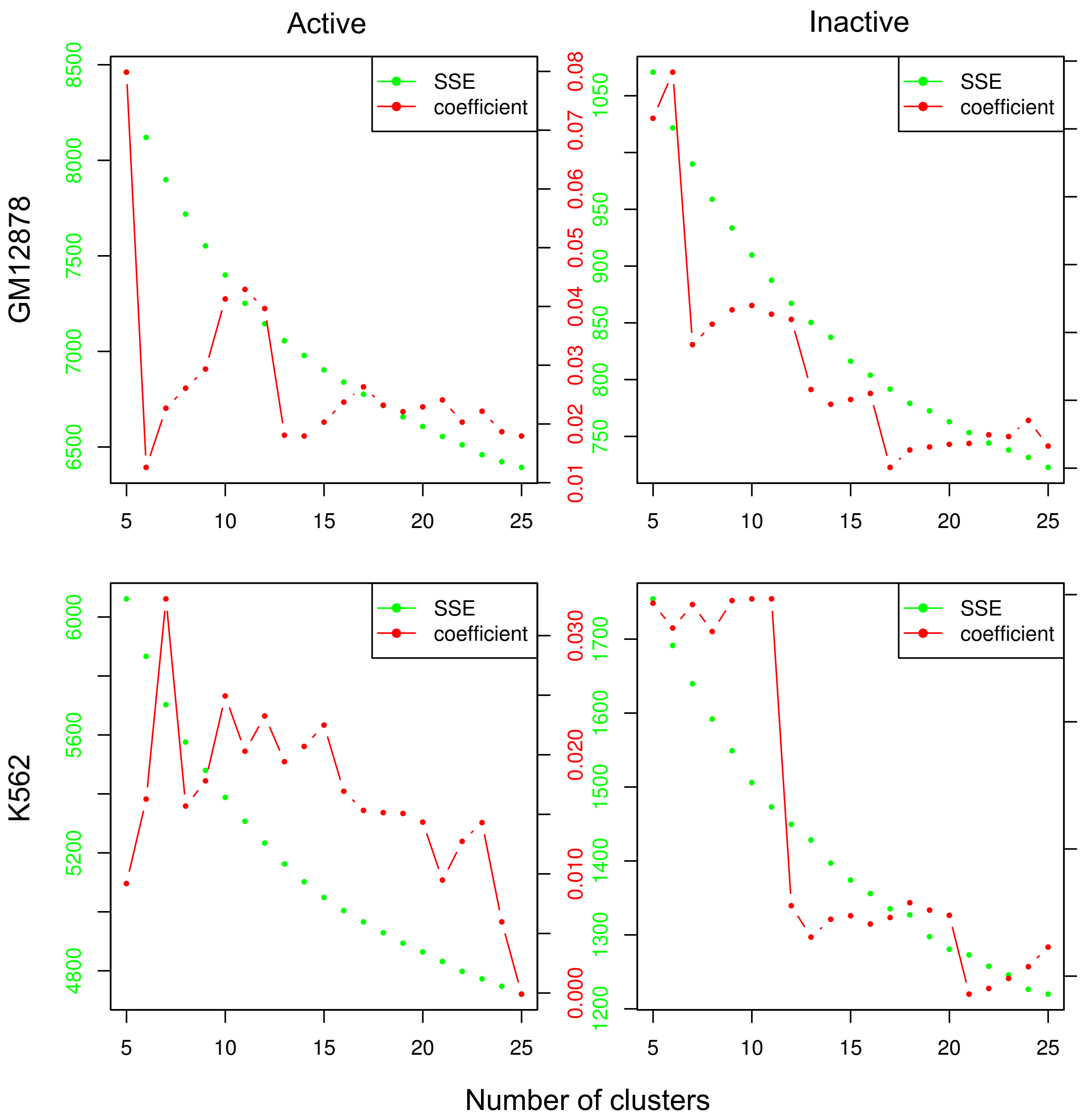

Figure S21: **Sum of square errors and coefficient in K-means clustering under different number of clusters, for active/inactive TSSs for GM12878/K562 cell line.** Plots show the sum of square error (green) and coefficient (red) in K-means clustering under different cluster number. Upper row: GM12878 cell line, bottom row: K562 cell line. Left column: active TSSs, right column: inactive TSSs. x axis refers to the number of cluster we choose for each run. green y-axis shows the sum of square error and the red y-axis shows the coefficient. When we choose the number of the clusters for final comparison, we choose the cluster number after sum of square error decrease significantly and he coefficient before dropping significantly. According to this, we choose cluster number as 12,12,10,12 for active/inactive TSSs in GM12878/K562 cell line, respectively.

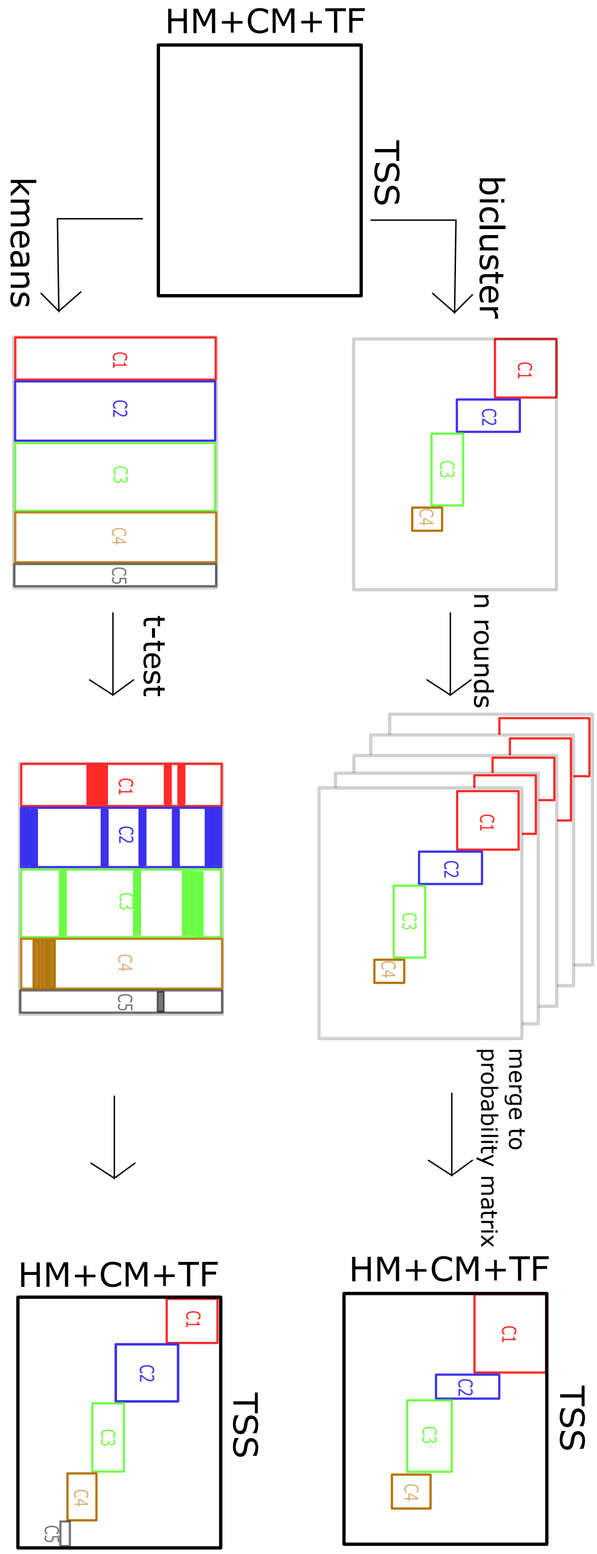

Figure S22: **Overview of biclustering methods.** The upper row of the sketch shows the SVD based biclustering procedure, while the bottom row depicts the alternative, k-means-based method. The SVD based method applies the s4vd repeatedly and obtains a biclustering with a probability for cluster membership. The k-means-approach first applies the k-means clustering algorithm to the matrix columns and subsequently uses the t-test to select rows thatbelong to column-clusters. (HM: histone modification. CM: chromatin modifier, TF: transcription factor).

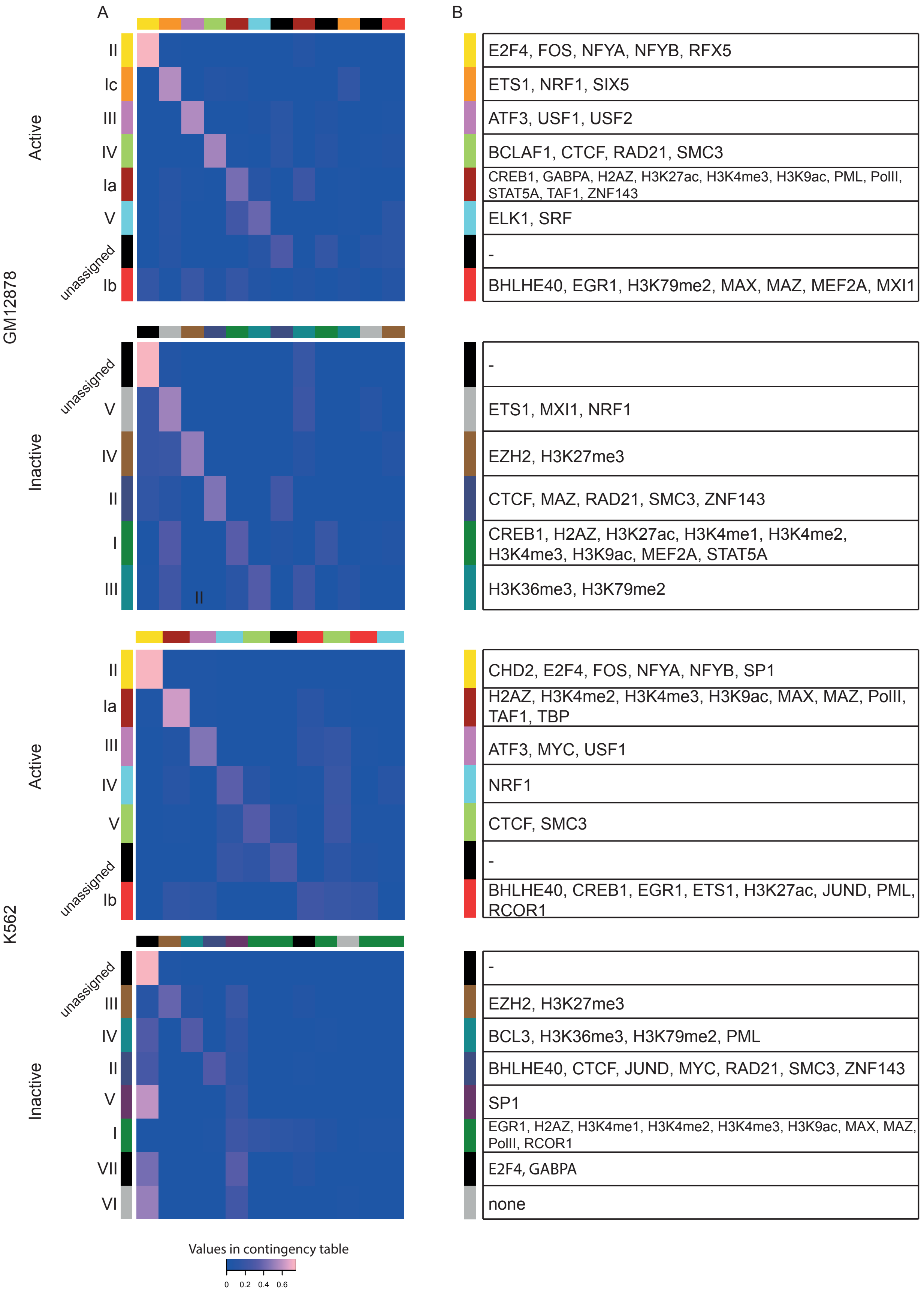

Figure S23: **Comparison of biclustering method and k means method.** TSSs clustering results from k means method are assign to biclustering using linear assignment algorithm. Heatmap for contingency table of two different cluster methods are shown in the left column. Color refers to the value in the contingency table, for active/inactive TSS in GM12878/K562 cell line, respectively. For each of the cluster in biclustering method, we show on the right column the ChIP-seq which overlaps with t.test result of k means clustering (described in the method).

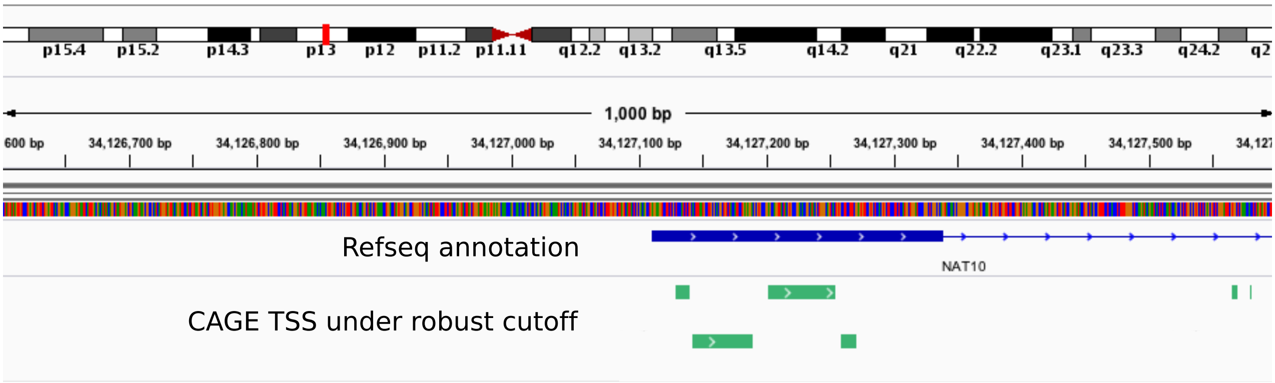

Figure S24: **Example of a promoter where multiple CAGE tag annotations are located.** Genome browser screen shot of the promoter of NAT10. There are 6 CAGE tag annotated promoters in +/-500bp window of its promoter.

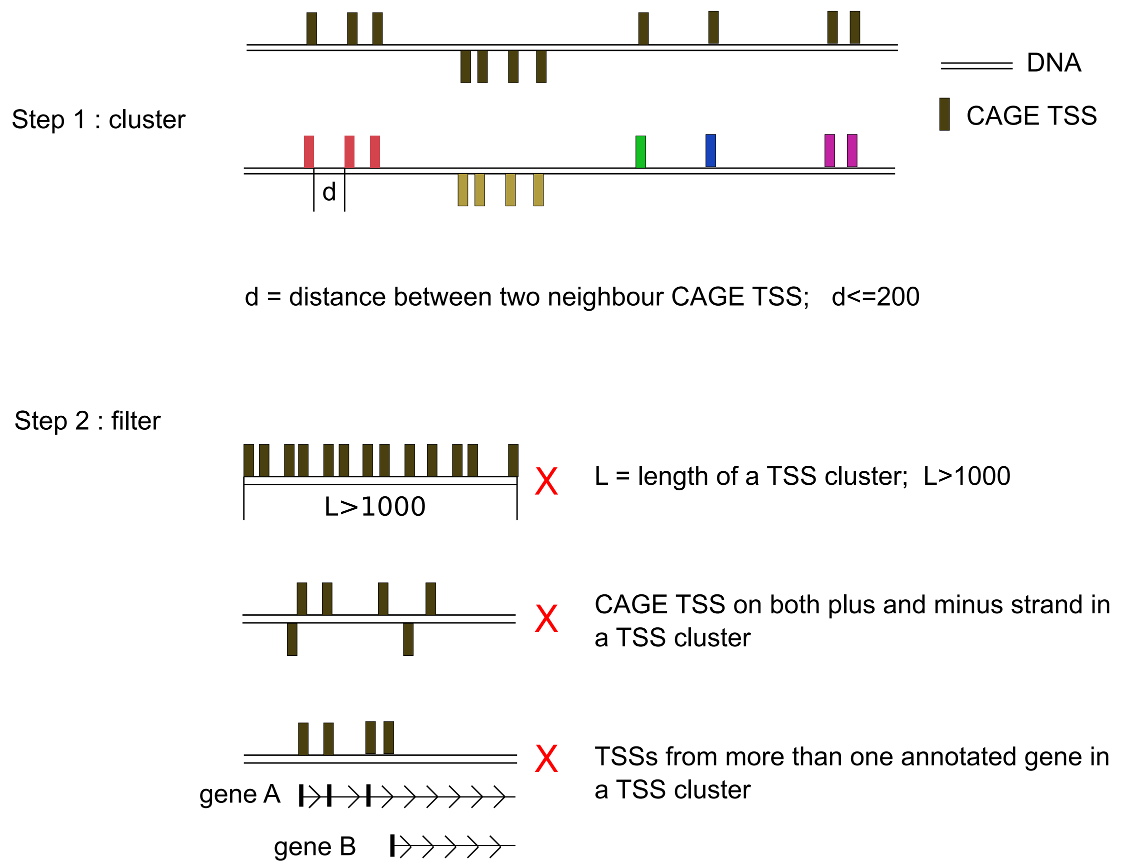

Figure S25: **Overview of definition of promoters based on CAGE tags.** From FANTOM 5 we import all CAGE tag annotation for a large number of cell-lines and cluster nearby peaks in the vicinity of genes into potential promoters. We treat the union of all these promoters across cell-lines as the set of potential promoters to study. Step 1 shows how we cluster CAGE tag annotated promoters to obtain less redundant CAGE-based promoters. Step 2 shows how we filter clusters from Step 1 to obtain more reliable CAGE-based promoters.
